# Biocrust Research in China: Recent Progress and Application in Land Degradation Control

**DOI:** 10.3389/fpls.2021.751521

**Published:** 2021-11-25

**Authors:** Xinrong Li, Rong Hui, Huijuan Tan, Yang Zhao, Rentao Liu, Naiping Song

**Affiliations:** ^1^Shapotou Desert Research and Experiment Station, Northwest Institute of Eco-Environment and Resources, Chinese Academy of Sciences, Lanzhou, China; ^2^Breeding Base for Key Laboratory Land Degradation and Ecological Restoration in Northwest China, Ningxia University, Yinchuan, China

**Keywords:** temperate desert, biocrust, soil eco-hydrology processes, land degradation control, nonvascular plant

## Abstract

Desert ecosystems are generally considered lifeless habitats characterised by extreme environmental conditions, yet they are successfully colonised by various biocrust nonvascular communities. A biocrust is not only an important ecosystem engineer and a bioindicator of desert ecological restoration but also plays a vital role in linking surficial abiotic and biotic factors. Thus, extensive research has been conducted on biocrusts in critical dryland zones. However, few studies have been conducted in the vast temperate deserts of China prior to the beginning of this century. We reviewed the research on biocrusts conducted in China since 2000, which firstly focused on the eco-physiological responses of biocrusts to species composition, abiotic stresses, and anthropological disturbances. Further, research on the spatial distributions of biocrusts as well as their succession at different spatial scales, and relationships with vascular plants and soil biomes (especially underlying mechanisms of seed retention, germination, establishment and survival of vascular plants during biocrust succession, and creation of suitable niches and food webs for soil animals and microorganisms) was analysed. Additionally, studies emphasising on the contribution of biocrusts to ecological and hydrological processes in deserts as well as their applications in the cultivation and inoculation of nonvascular plants for land degradation control and ecological restoration were assessed. Finally, recent research on biocrusts was evaluated to propose future emerging research themes and new frontiers.

## Introduction

The term biological soil crust or biocrust was first used in the 1950s (Belnap, 2003) and is characterised by a complex consortium of cyanobacteria, green algae, lichens, mosses, and other microorganisms associated with surface soil particles, cemented *via* mycelia, rhizoids, and secretions (West, 1990; Li, 2012). Biocrust is a major land cover type in arid and semiarid regions worldwide (Eldridge and Greene, 1994), currently covering approximately 12% of Earth’s terrestrial surface (Rodriguez-Caballero et al., 2018). However, research in this regard is limited and has been conducted only for a few climatic regions. Studies on biocrusts have been traditionally conducted by researchers from a few countries (e.g., the United States, Australia, Israel, Germany, Spain, and Mexico; Belnap and Lange, 2003). It is striking that regions identified as being some of the most densely covered by biocrusts are also the least studied (for example, the large deserts in Asia; Rodriguez-Caballero et al., 2018).

**Figure 1:**
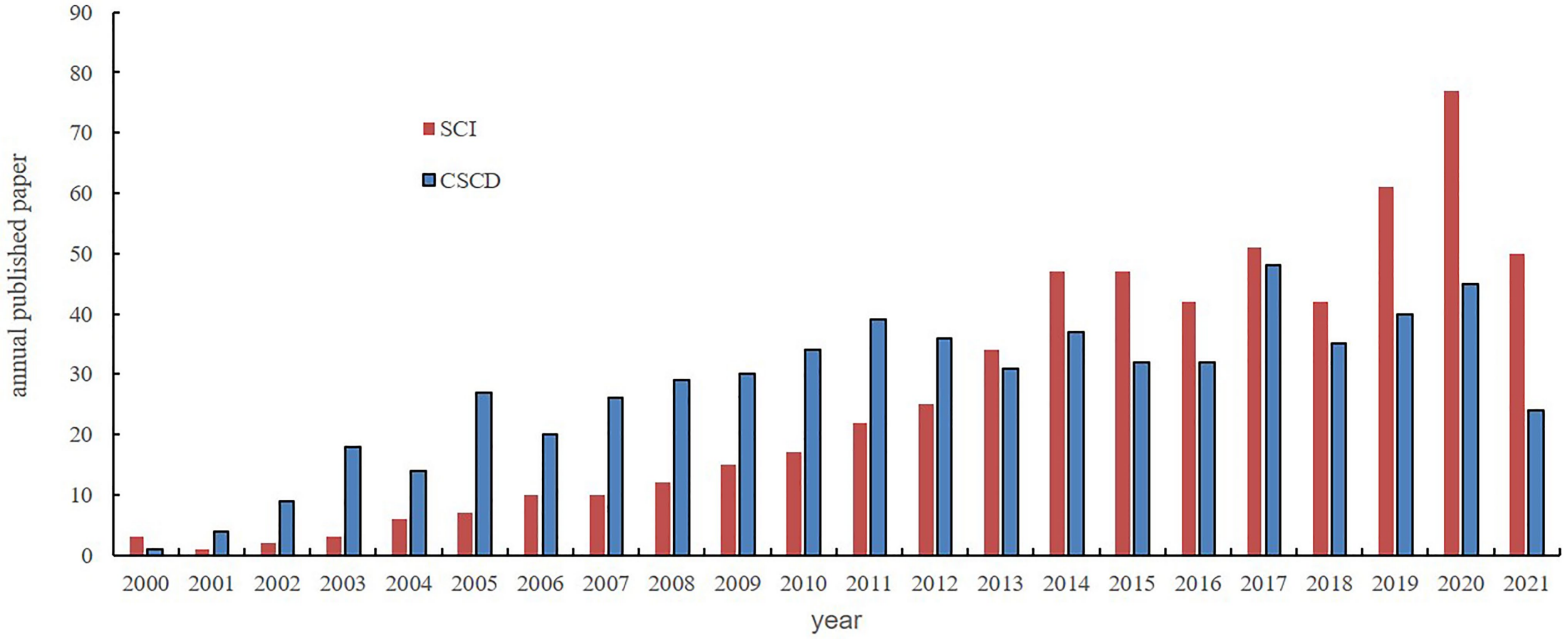
Publications on biocrusts in the last two decades in China by subject search (biological crust, cryptogamic crust, biocrust, microbiotic crust, microphytic crust, and microbial crust) and search date (January, 1995~August, 2021; SCI indicates papers published on the international journals, included in Science Citation Index; CSCD indicates papers published in Chinese, included in Chinese Science Citation Database. The search report was completed by Lanzhou novelty search consulting Center, Chinese Academy of Sciences, www.llas.cas.cn).

Recently, biocrust research has become a global endeavour and several research groups in this regard have emerged in countries such as China (Li et al., 2016a). The scientific community in China has indicated an increasing interest in biocrust research over the last two decades (Figure 1). In particular, recent studies have focused on the ecosystem multifunctionality of biocrusts (Su et al., 2020). Specifically, most studies have been conducted on the formation, structures, community compositions, succession, spatiotemporal distributions, and ecohydrological functions of biocrusts at different scales; moreover, the application of artificially cultivated biocrusts in land degradation control such as fixation of dune extension, and biocrust responses to climate change and various other disturbances since the late 1990s have also been studied (Li et al., 2012, 2017). This paper assessed the progress in biocrust studies conducted in China since 2000. Additionally, novel insights and future research hotspots were summarised. We conclude that these studies not only compensate for the lack of biocrust studies in temperate desert regions but also improve our limited quantitative understanding of nutrient cycling, carbon cycling, and water balance in drylands, enhance the universality of our conclusions on biocrusts, and provide relevant information for future ecosystem management and ecological restoration in arid and semiarid regions worldwide.

## Biocrust Formation, Composition, Successional Dynamics, and Controlling Factors in Temperate Deserts From China

### The Formation and Structure of Biocrusts

**Figure 2:**
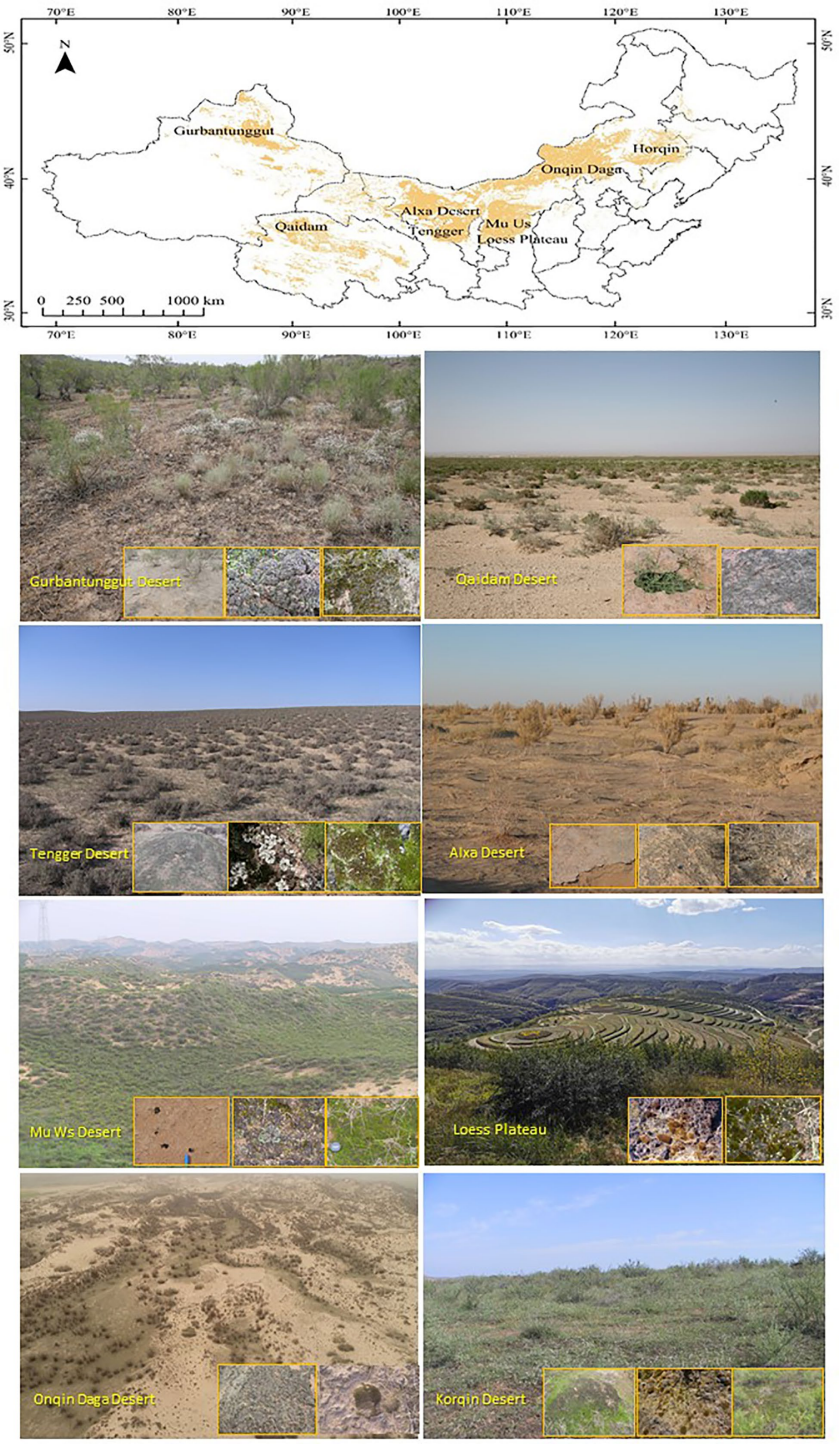
The main research sites and types of biocrust of Chinese deserts and the Loess Plateau.

Chinese temperate deserts are distributed mostly in northwest China, roughly on the west of 108°E and the north of 36^o^N, involving Xinjiang, Qinghai, Gansu, Ningxia, and Inner Mongolia (Figure 2). The climates vary from extreme arid to arid to semi-arid, and from temperate to warm temperate, the annual precipitation ranges from 30 to 400mm from the west to east of the country. Phytogeographically, this floristic division belongs to the Central Asian sub-region, the Sahara-Gobi floristic region (Wu, 2021). Unlike hot and cold deserts, the higher species richness of biocrust communities in Chinese temperate deserts is characterised by complex patchy distributions of cyanobacteria, lichens, and mosses; additionally, these deserts are particularly rich in lichens and mosses even at small spatial scales (Li, 2012; Li et al., 2017). Scanning electron microscopy results have indicated that biocrust keystone component such as cyanobacteria, lichens, and mosses *via* the filaments, fungal hyphae, rhizoids, and extracellular polymer secretions bind the finer particles of surface soil, thus forming unique biocrust structures (Hu et al., 2002; Zhang, 2005; Zhang et al., 2006, 2013, 2014; Gao et al., 2017a). The vertical distributions of cyanobacteria and microalgae in biocrusts have been distinctly laminated into inorganic (0–20μm), algae-dense (20–1,000μm), and algae-sparse (1,000–5,000μm) layers at the micro-scale (Hu et al., 2013). The primary cementing pattern that sustains the biocrust structure changes with the succession of the biocrust, thus implying that the cohesive role of extracellular polymeric substances in cementing the soil particles is later strengthened by cyanobacteria, desert algae filaments, fungal hyphae of lichens, and moss rhizoids (Hu and Liu, 2003; Zhang et al., 2007).

### The Succession and Species Composition of Biocrusts

At the initial stage of biocrust formation, increasing dust deposition on topsoil triggers the colonisation and development of biocrusts (Li et al., 2004a, 2010a). Long-term monitoring of sand-binding vegetation in the Shapotou region of the Tengger Desert has indicated that physical crusts characterised by high clay and silt concentrations are formed due to dust and silt deposition on the sand surface (Li et al., 2004a,b). Further, dust sinking and precipitation affect the early period of sand stabilisation through revegetation. Subsequently, bacteria, fungi, actinomycetes, and cyanobacteria colonise the surfaces and sub-surfaces of stabilised dunes. In this process, the shifting of soil microbial community functional gene structure plays key roles in driving the biocrust colonisation and development (Liu et al., 2017; Hu et al., 2019).

Biocrust is primarily composed of cyanobacteria, green algae, diatoms, and euglenoids in the early-successional stages, with cyanobacteria being the dominant species (Hu et al., 2004; Li et al., 2004a). A total of 121, 23, 21, 23, and 56 algal species have been identified in the biocrust communities of the Gurbantunggut Desert, the Qaidam Basin, the Alxa-Tengger Desert, Horqin Sandland, and the Kubuqi Desert, respectively. In particular, *Microcoleus vaginatus* (Vauch.) Gom. was found to be the dominant species (Hong et al., 1992; Li et al., 2004a; Zhang et al., 2009a, 2011a, 2016a; Hu et al., 2013). However, only 11 cyanobacteria and algae species were identified in the biocrust communities of the Loess Plateau; specifically, the commonly occurring *M. vaginatus* has not been recorded so far (Reynaud and Lumpkin, 1988). In contrast to other deserts worldwide, the Gurbantunggut Desert exhibits a high diversity of cyanobacterial and microalgal morphotypes (Zhang et al., 2011a). Additionally, bacteria, fungi, and Archaea significantly contribute to biocrust formation during early successional stages (Zhao et al., 2020a). The investigation of the microbial functional potentials of biogeochemical processes during biocrust development indicated that fungi are the key microbial mediators in C and N cycling for late successional biocrusts, the bacterial community was the major contributor to the P and S cycles (Qi et al., 2021), and microbial functional structure may be a potential indicator of soil restoration and land degradation control (Grishkan et al., 2015; Liu et al., 2017, 2018; Zhao et al., 2020b).

When lichens are dominated species at the later successional stage of biocrust, the following new species such as *Bacidia heterochroa* (Müll. Arg.) Zahlbr, *Porina aenea* (Wallr.) Zahlbr., *Buellia alboatra* (Hoffm.) Branth, *Buellia venusta* (Körb.) Lettau (I, VI), *Endocarpon deserticola* sp. nov., *Endocarpon unifoliatum* sp. nov., *Fulgensia desertorum* (Tomin) Poelt, *Rinodina bischoffii* (Hepp) A. Massal, and *Seirophora orientali* have been identified in the Tengger and Gurbantunggut deserts (Liu, 2012; Yang and Wei, 2014; Zhang et al., 2017). Moreover, *Collema tenax* (Sw.) Ach., *Lecidea decipiens* (Hedw.) Ach., *Xanthoparmelia deserborum* Hale., and *Diploschisttes muscorum* (Scop.) R. Sant are the dominant species in the stabilised sand dunes (Zhang et al., 2007).

Finally, moss-dominated crusts form on dune surfaces and likely improve the fertility and water-holding capacity of topsoil (Li et al., 2002, 2003, 2004a, 2007a). In contrast to other deserts and sandlands in China, the biocrust communities in Mu Us and Horqin sandlands exhibit a relatively higher coverage and diversity of mosses (Guo et al., 2008; Liu et al., 2017), because evident positive correlations between moss diversity and precipitation have been found along precipitation gradients (Li et al., 2017). Sixteen moss species have been reported in the stabilised sand dunes of the Tengger Desert, with *Bryum argenteum* Hedw. being the dominant species (Li et al., 2010a). The Gurbantunggut Desert indicates a lower moss diversity with *Bryum argenteum* Hedw., *Bryum capillare* Hedw., *Grimmia anodon* Bruch & Schimp, and *Grimmia pulvinate* (Hedw.) Sm. being the dominant species (Li et al., 2004b; Zhang et al., 2007). Thus, biocrusts in temperate deserts are classified into “cyanobacteria and algae dominated, lichen dominated, lichen-moss dominated, and moss-dominated crusts” (Li et al., 2003; Lan et al., 2012; Zhang and Zhang, 2014).

### The Controlling Factors for Biocrusts Distribution

The primary factors determining the spatial distributions of biocrusts at different scales have also been elucidated. Surface micro-geomorphological features such as small soil mound, and the hollow, crest, windward slope as well as leeward slope of fixed dune determine the community diversity of biocrusts at the micro-scale (Li et al., 2002, 2010a). Micro-geomorphology has created various habitats at a small-scale affecting spatial distribution of nonvascular plants by reallocating related abiotic resources (Li et al., 2012). Further, the cover and diversity of biocrusts are significantly influenced by dust deposition, light, soil moisture, and soil nutrients at the small and medium scales (Li et al., 2010a; Zhang et al., 2015). The accumulation of dust deposition on fixed dune surface is one of the prerequisites for the colonisation and development of cyanobacteria crust in the initial successional stage (Li et al., 2000). Shade and higher surface soil moisture under shrub canopy enhances moss covering and species richness at the small scale (Li et al., 2010a), strong light exposure and stable surface soil with higher nutrient content are favourable for lichen development (Guo et al., 2008; Li et al., 2017). Finally, precipitation, physiochemical properties of topsoil, and distribution of vegetation cover primarily determine the spatial distributions of dominant species in biocrust communities at the landscape (desert regions of northern China, Figure 2), regional (specific desert regions), and local (specific sample plots) scales, respectively (Li et al., 2017).

## Biocrusts Response to Abiotic Stresses and Climate Change

### The Response to Abiotic Stresses

Although organisms that form biocrusts can survive in extreme environments, they are sensitive to global climate change as well as other stresses (Li et al., 2018), including the physio-ecological responses of biocrusts to variations factors such as precipitation, UV-B radiation, nitrogen, salinity, temperature, and light. Biocrusts can maintain physiological activity by utilising limited rainwater (1mm), dew, and snowmelt (Rao et al., 2009; Zhang et al., 2009b, 2011b; Wu et al., 2012, 2013; Li et al., 2014a,b; Gao et al., 2017b; Hui et al., 2021). Winter snowfall can stimulate the nonvascular plants in biocrusts to produce higher photosynthetic and respiratory rates (Su et al., 2013a; Hui et al., 2016a; Yin and Zhang, 2016; Zhao et al., 2016a). Further, *Syntrichia caninervis* exhibits an upside-down water collection system (Tao and Zhang, 2012; Wu et al., 2014; Pan et al., 2016). It is interesting that drought induced dormancy (inactive) is another strategy to protect biocrusts from UV-B radiation (Hui et al., 2016b), high temperatures (Lan et al., 2014a), and salt stresses (Lan et al., 2010).

It should be noted that enhanced UV-B radiation significantly decreases the photosynthetic activity and growth rate of algae and induces cellular oxidation and DNA damage (Wang et al., 2008a, 2012; Chen et al., 2009; Xie et al., 2009). Specifically, UV-B radiation inhibits the net photosynthetic rate of algae *via* indirect (decreased chlorophyll concentration) and direct (changed the structure of photosynthetic proteins) mechanisms; however, algae can alleviate the detrimental effects of UV-B radiation on photosynthesis and DNA by relying on exogenous chemicals (ascorbic acid, N-acetylcysteine, and extracellular polymers; Wang et al., 2008a, 2012; Xie et al., 2009). Similarly, increased intensity and exposure of UV-B radiation can significantly inhibit the photosynthetic rate of biocrust mosses (Wu et al., 2005; Xue et al., 2005) and cause cell membrane damage, thus resulting in dysregulation of antioxidant enzymes (Hui et al., 2014, 2015). Increased UV-B radiation can also damage the cells and chloroplast ultrastructures of mosses (Hui et al., 2013). However, biocrust organisms have developed a series of defence mechanisms against UV-B radiation such as avoidance, accumulation of UV-B-absorbing compounds, and DNA damage repair (Wang et al., 2010; Chen et al., 2012, 2013; Ma et al., 2012; Hui et al., 2014). In addition, damage by enhanced UV-B radiation on mosses *Bryum argenteum* and *Didymodon vinealis* might be alleviated by water deficit (Hui et al., 2018).

Biocrust algae can endure and resist salt stresses (Tang et al., 2007). Specifically, salt stresses can lead to the synthesis of polysaccharides through changes in carbohydrate metabolism and exogenous polysaccharides can subsequently increase salt tolerance (Chen et al., 2003, 2006a). Algae can adapt to high temperatures and high light intensities, thus promoting the synthesis of polysaccharides (Ge et al., 2014a,b). Further, high temperatures accelerate the N-fixing activities of algae and lichen crusts, thus facilitating N fixation by biocrusts (Zhang et al., 2012a). Moreover, low temperatures and dark conditions allow biocrust recovery, while high light intensities inhibit recovery (Lan et al., 2015). The observation of chlorophyll fluorescence and CO_2_ exchange under a series of photosynthetically active radiation (PAR) gradients indicated that acclimation to high PAR resulted in a special structure and significantly high accumulation of photosynthetic pigments in lichen crusts (Wu et al., 2017).

### The Response to Climate Change

Experimental results have indicated that simulated nitrogen (N) deposition significantly affected the biomass, carbon and N metabolism, osmotic adjustment substances, and antioxidant enzyme activities of biocrusts (Zhang et al., 2016b). Low rates of N addition have been shown to exert a positive effect on the growth and physiological activity of moss crusts. Contrarily, high rates of N addition exert evident negative effects. Specifically, positive effects are weakened with increasing N concentrations (e.g., addition of 1g N m^−2^ a^−1^ to algae and lichen crusts); further, decreased positive effects were observed in a moss crust subjected to 0.3g N m^−2^ a^−1^, thus resulting in negative effects (Zhou et al., 2016; Zhang et al., 2016b). In particular, the addition of inorganic N can significantly alter the diversity and community structure of microbes in biocrusts (Wang et al., 2015).

Warming and rainfall reduction can alter the community compositions, structures, and characteristics of biocrusts, which further affect the sustainable development of desert ecosystems (Li et al., 2016a, 2018). Meanwhile, warming and different types of precipitation events in biocrust-dominated desert ecosystems impact soil carbon release through changes in the magnitude of soil respiration (Guan et al., 2021). Long-term warming and reductions in precipitation influenced the moss-dominated biocrust *via* a decrease in moss cover and biomass, even causing a decrease in moss species richness, while the lichen-dominated biocrusts did not respond to warming and drought. Divergent responses of the dominant species in biocrust communities could increase probability to partly maintain the multifunctionality of biocrusts in arid desert ecosystem (Li et al., 2021a).

## Biocrusts Serve as Ecosystem Engineers

### The Contributors to Soil Stability and Habitat Improvement

Biocrust can significantly enhance the resistance of soil surfaces to wind erosion by increasing the wind friction velocity threshold of soils (Wang et al., 2009a; Bu et al., 2015a). The viscous thalli, slime and tailpieces coupled with filaments of actinomycetes and fungi, are responsible for binding together sand particles and thus forming tough cortical crusts on sandy surfaces (Li et al., 2004a). Wind erosion rates for sandy soil with 0% crust cover was about 46, 21, and 17 times the soil with 90% crust cover at wind velocities of 18, 22, 25m s^−1^, respectively (Zhang et al., 2006). Wind and water erosion rate decrease with biocrust development from initial cyanobacteria dominated to the later lichen and moss dominated crusts *via* promoting shallow soil aggregate structure, organic matter, water-holding capacity, and biocrust thickness, cover, as well as biomass (Li, 2012). Biocrusts should be strongly protected to avoid exacerbating wind and water erosion in dryland (Zhao et al., 2014a; Bu et al., 2015a). Higher cover of moss has an effective ability to control soil water erosion in the Loess Plateau, based on a threshold moss cover of 35% beyond which water erosion was completely prevented (Gao et al., 2020a), because biocrusts inhibited runoff erosion through direct physical protection related to biocrust cover and biomass and through the indirect modification of soil properties (Gao et al., 2020b), in particular, decreased raindrop erosivity (Zhao and Xu, 2013; Zhao et al., 2014a).

Colonisation and development of biocrusts are important indicators of soil ecological health in deserts and sandy lands (Li et al., 2016a). Biocrusts promote topsoil formation on sand surfaces and improve the physicochemical and biological properties of topsoil (Chen and Li, 2012; Zhao and Xu, 2013; Chen and Duan, 2015; Li et al., 2017; Niu et al., 2017). A comparison of biocrust covered shallow soil indicated that the clay content increased from 3.0 to 5.0% during the initial successional stage to 8.0–25.0% during the late successional stage; moreover, the soil exhibited aggregation (>250μm; Li et al., 2007b; Chen et al., 2008; Guo et al., 2008; Gao et al., 2010, 2012, 2017a; Zhang et al., 2014a) and significantly increased organic carbon content, total nitrogen, total phosphorus, and total potassium (Li et al., 2002, 2013a, 2016b; Gao et al., 2017a). Further, biocrusts can promote the accumulation of fine particles and nutrient enrichment of topsoil through corrosion of sand surface minerals and deposition of wind and water eroded substances, thus promoting soil formation and fixing sand dune surface (Li et al., 2013a; Liu et al., 2016a; Gao et al., 2017a). Additionally, biocrusts have been shown to enhance the activities of soil ureases, invertases, catalases, and dehydrogenases (Zhang et al., 2012b; Liu et al., 2014; Zhou and Zhang, 2014; Hu et al., 2016).

### The Roles in C and N Cycling

Biocrusts significantly participate in the carbon and nitrogen cycles of desert ecosystems; thus, they are an important source of organic carbon and nitrogen in soils (Li, 2012; Su et al., 2013b; Wu et al., 2015; Zhao et al., 2016b). Carbon release from biocrusts increases with increasing total precipitation and snowfall *via* increasing respiration (Hui et al., 2016a; Zhao et al., 2016a), meanwhile temperature increases significantly affect the biocrust carbon budget. A temperature increase of 2.5°C significantly inhibits the photosynthetic rates of biocrusts to consequently increase the carbon release rates (Huang et al., 2014a; Ouyang and Hu, 2017). Specifically, soil moisture and effective wetting time determine the amount of carbon sequestration by biocrusts (Li et al., 2018, 2021a). The carbon fixation is higher with high-frequency rainfall, even if the total amount of seasonal rainfall was the same (Huang et al., 2014a). Compared with cyanobacteria crusts, lichen and moss soil crusts had the higher photosynthetic activities (Fv/Fm), and about 2.4–7.5-fold higher than the former (Lan et al., 2017). The range of optimal gravimetric water content for early biocrusts to fix carbon was 1–3.5%, and 1–5% for the later successional biocrusts. The annual carbon fixation was 11.36g C m^−2^ yr^−1^ for cyanobacteria-algae dominated crusts and 26.75g C m^−2^ yr^−1^ for lichen-moss dominated crusts. These findings indicate the recovery of biocrusts is expected to significantly increase carbon input into sandy desert ecosystems (Li et al., 2012). In addition to these biocrust nonvascular plants, in the C cycle, bacterial and fungal functional genes in biocrust communities were involved in the degradation of labile and recalcitrant C, suggesting that bacteria and fungi cooperate in C degradation (Zhao et al., 2020b). However, daily net carbon fluxes in the biologically crusted soils and bare land showed carbon release at most times and total carbon production ranged from 48.8–5.4g C m^−2^ yr^−1^ to 50.9–3.8g C m^−2^ yr^−1^ (Su et al., 2013a).

The nitrogen fixation ability of biocrusts ranges between 2.5 and 62.0μmol C_2_H_4_ m^−2^ h^−1^ (Wu et al., 2009; Su et al., 2011). Among the different biocrusts, algae crusts exhibit the highest average nitrogen fixation activity (28.1μmol C_2_H_4_ m^−2^ h^−1^), followed by lichen (24.3μmol C_2_H_4_ m^−2^ h^−1^) and moss (14.0μmol C_2_H_4_ m^−2^ h^−1^) crusts (Wu et al., 2009; Su et al., 2011). The annual nitrogen fixation activity of biocrusts ranges between 3.7 and 13.2mg m^−2^ a^−1^ (Wu et al., 2009; Su et al., 2011). Further, the nitrogen mineralisation rates (nitrate nitrogen, ammonium nitrogen, and inorganic nitrogen) of moss crusts (0.14–0.83mg kg^−1^ d^−1^) are higher than those of algae crusts (0.06–0.58mg kg^−1^ d^−1^; Hu et al., 2015). These results provided evidence that biocrusts can add nitrogen to desert ecosystems, transform nitrogen into soil nutrients, and directly supply N to eremophytes (Wu et al., 2009; Zhao et al., 2010a; Su et al., 2011; Hu et al., 2014). Nitrogen fixation exhibits a significant positive correlation with mineralisation and precipitation, and different biocrust types indicate significantly different responses to nitrogen increases (Hu et al., 2014; Liu et al., 2016b). In addition, factors affecting carbon cycles also affect nitrogen cycles (Wu et al., 2009; Su et al., 2011; Hu et al., 2015). It should be noted that moderate pasturing can promote nitrogen fixation by biocrusts (Liu et al., 2009). It has been explored that biocrusts and vegetation patches present a “source-sink” relationship for carbon and nitrogen at the desert landscape scale (Li et al., 2008a), suggesting that biocrust patches significantly contribute to maintaining and managing the C and N levels in vegetation patches (Li et al., 2013a; Zhao and Xu, 2013; Liu et al., 2016a). These findings implied that the conversion of carbon and nitrogen “source-sink” relationships can be mediated through desert ecosystem management (Li et al., 2013a).

### Biocrusts Mediated Soil-Water Balance

Biocrusts significantly affect the soil hydrological processes in deserts and sandy lands by altering rainfall infiltration, runoff, surface evaporation, non-rainfall water collection (dew, fog, and water vapor sorption) as well as the moisture of shallow and deep soils (Liu et al., 2006; Zhang et al., 2008, 2009b; Li et al., 2010b, 2021a,b; Pan et al., 2010; Xiao et al., 2010; Bu et al., 2015a; Wang et al., 2017).

Biocrusts significantly alter the spatiotemporal redistributions of rainfall infiltration and soil moisture as well as reduce the effective supplementation of rainfall to deep soil (Li et al., 2001; Bu et al., 2015a; Wang et al., 2017; Xiao et al., 2019). The Gurbantunggut Desert receives precipitation ranging from 70 to150 mm; further, moss-, lichen-, and algae-dominated crusts have been shown to reduce the infiltration rate by 16.50–36.10, 33.98–46.42, and 35.92–50.39%, respectively, while reducing the 1-h accumulated infiltration rate by 16.10, 28.56, and 26.56%, respectively (Zhang et al., 2006). The precipitation in Tengger Desert ranges from 150 to 200 mm and the infiltration intercepted by biocrusts exhibits the following order: moss crust>lichen crust>algae crust. The three different biocrusts presented no significant differences when the precipitation was less than 5mm or greater than 10mm (Li et al., 2010b). The biocrusts in Mu Us and Horqin sandy lands (annual precipitation=300–500mm) reduce both infiltration rates and infiltration depths (Bu et al., 2013, 2015b). Further, biocrusts have been shown to reduce infiltration rates in the Loess Plateau area (annual precipitation=450mm), thus resulting in shallow distributions of soil moisture and increased surface runoff (Xiao et al., 2011, 2016; Zhao and Xu, 2013; Zhao et al., 2014b).

Biocrusts can reduce the occurrence of surface runoff and soil erosion by absorbing the energy produced by splashing raindrops (Xiao et al., 2011; Zhao et al., 2014a). Scanning electron microscopy results have indicated that sandy soils are sufficiently porous for water flow (Wang et al., 2017). However, mud and clay particles in the crustal layer expand upon wetting and consequently inhibit soil moisture infiltration. Further, certain cyanobacteria can rapidly expand in response to rainfall, thus closing the water flow paths on the soil surface. Contrarily, certain well-developed moss-crust surfaces are difficult to saturate with water, thus allowing water infiltration to deep soil. It has been noted that biocrusts with *Endocarpon pusillum* Hedw. and *Collema tenax* can intercept rainfall infiltration, while those with *Psora decipens* (Hedwig) Hoffm and *Toninia* sp. are conducive to rainfall infiltration due to mesh cracks on the surface (Wei, 2005). We utilised the Limburg soil erosion model (LISEM) to conclude that the algae crust-covered leeward slope of a sand dune was more likely to generate flow than the moss crust-covered windward slope of a sand dune (Li et al., 2001). Further, long-term experiments and simulations have indicated that the relationship between biocrusts and precipitation infiltration primarily depends on biocrust characteristics (porosity, thickness, and species composition), topsoil properties (initial soil moisture content and texture composition), and local precipitation characteristics (raindrop diameter, rainfall duration, and rainfall intensity; Li et al., 2001, 2002).

Biocrusts affect surface evaporation by altering the physicochemical properties of soils (Zhang et al., 2008; Xiao et al., 2010). Specifically, biocrusts promote evaporation by reducing the surface reflectance and increasing the water-holding capacity of topsoil (Bu et al., 2013). It has also been reported that biocrusts reduce evaporation by closing the soil surface (Wang et al., 2005). Further, the effects of biocrusts on surface evaporation are influenced by regional climatic conditions (Zhang et al., 2008), soil moisture status (Liu et al., 2007), microtopography (Li et al., 2010a), and biological characteristics of biocrusts (Wang et al., 2017). Additionally, different biocrust types and coverage rates exert different effects on surface evaporation (Sun et al., 2008; Xiao et al., 2010). For instance, moss-dominated crusts first promote and then inhibit evaporation, thus ensuring that moisture remains in the topsoil for a prolonged duration; therefore, moss-crusts, which exhibit the highest water-holding capacity, are significant for the germination and establishment of therophytes (Li et al., 2003, 2004b, 2005; Su et al., 2007; Zhang et al., 2008).

Non-rainfall water such as dew is not only a significant water resource for non-vascular plants and other tiny organisms in biocrusts but also affects the activities of these species (Rao et al., 2009; Li, 2012; Huang et al., 2014b; Jia et al., 2014; Liu et al., 2016a; Ouyang and Hu, 2017). Long-term monitoring on dew entrapment in the Tengger Desert has indicated that the mean daily amount of dew on the surfaces of moss- and algae-crusts is approximately 0.15mm d^−1^, with a maximum value of ~0.50mm d^−1^. The total amount of condensed water in shifting sands, physical crusts, and biocrusts accounts for 15.9, 22.9, and 37.9% of the concurrent precipitation, respectively (Pan et al., 2010). The daily amounts of dew on the surfaces of moss-, algae-, and lichen-crusts in the Gurbantunggut Desert were 0.14, 0.11, and 0.09mm d^−1^, respectively (Zhang et al., 2008). However, the mean daily amounts of dew on the surfaces of moss- and algae-crusts in the Mu Us Sandy Land were 0.12 and 0.10mm d^−1^, respectively (Sun et al., 2008). The dew amount of the biocrusts was increased by up to 130.5% on the loess and 157.1% on the aeolian sand in semiarid regions (Li et al., 2021b). Non-rainfall water forms on biocrusts owing to their surface microclimates (Liu et al., 2007; Zhang et al., 2009b), adherence to several microbial organic components (Pan et al., 2010), trichome development, and the special water collection and transmission systems (grooves and verruca) of mucilage secretions (Rao et al., 2009) and leaf tips (Tao and Zhang, 2012). Further, nocturnal absorption of condensation can compensate for diurnal moisture losses from soil surfaces, which is conducive to the retention of surface moisture by biocrusts (Pan et al., 2010; Pan and Wang, 2014). Consequently, unlimited reductions in surface moisture are prevented during the dry season (Li et al., 2014c). Biocrusts are associated with much greater non-rainfall water deposition capacity, and change non-rainfall water distribution along with soil depth, implying that they play a critical role in surface soil water balance of dryland ecosystems (Li et al., 2021b,c).

In ecological restoration practice of China such as establishing artificial sand-binding vegetation to protect cropland, settlement and transportation route from sand burial, the high biocrust cover is not conducive to shrub planting in high density on sand dunes because biocrusts reduce the moisture of deeper soil by reducing infiltration (Li et al., 2004a, 2010b; Xiao et al., 2016; Xiao and Hu, 2017). However, the redistribution of water and nutrients from biocrust patches to plant patches can be crucial in the maintenance of vegetation productivity in natural desert landscape (Li et al., 2008a). Thus, maintaining a stable sink-source relationship between biocrust patches and plant patches is beneficial to the water balance of desert ecosystems (Li et al., 2009, 2016a). However, global warming affects these hydrological roles of biocrusts, for example, reduces dew formation, weakens infiltration interception and increases evaporation, finally altering the hydrological processes and original water balance of desert ecosystems (Li et al., 2018, 2021a).

### Effects of Biocrusts on Vascular Plant and Soil Biomes

Land surface in arid and semiarid regions is often characterised by mosaic patches of biocrusts and vascular plants due to limited water availability (Li, 2012). Biocrusts are beneficial for the survival and reproduction of vascular plants, since they increase N uptake in adjacent vascular plants and promote carbon uptake in C_3_ plants as demonstrated by isotope tracing (Zhao et al., 2010b). Cyanobacteria- and moss-crusts significantly increase the germination and survival rates of annual plants (Su et al., 2007, 2009). However, other studies have concluded that vascular plant seeds are not retained on the smooth moss-crust surfaces in windy environments, thus indirectly reducing the likelihood of seed germination (Li et al., 2005). In addition, an increase in vegetation cover and surface litter can be detrimental to biocrusts (Guo et al., 2008).

**Figure 3:**
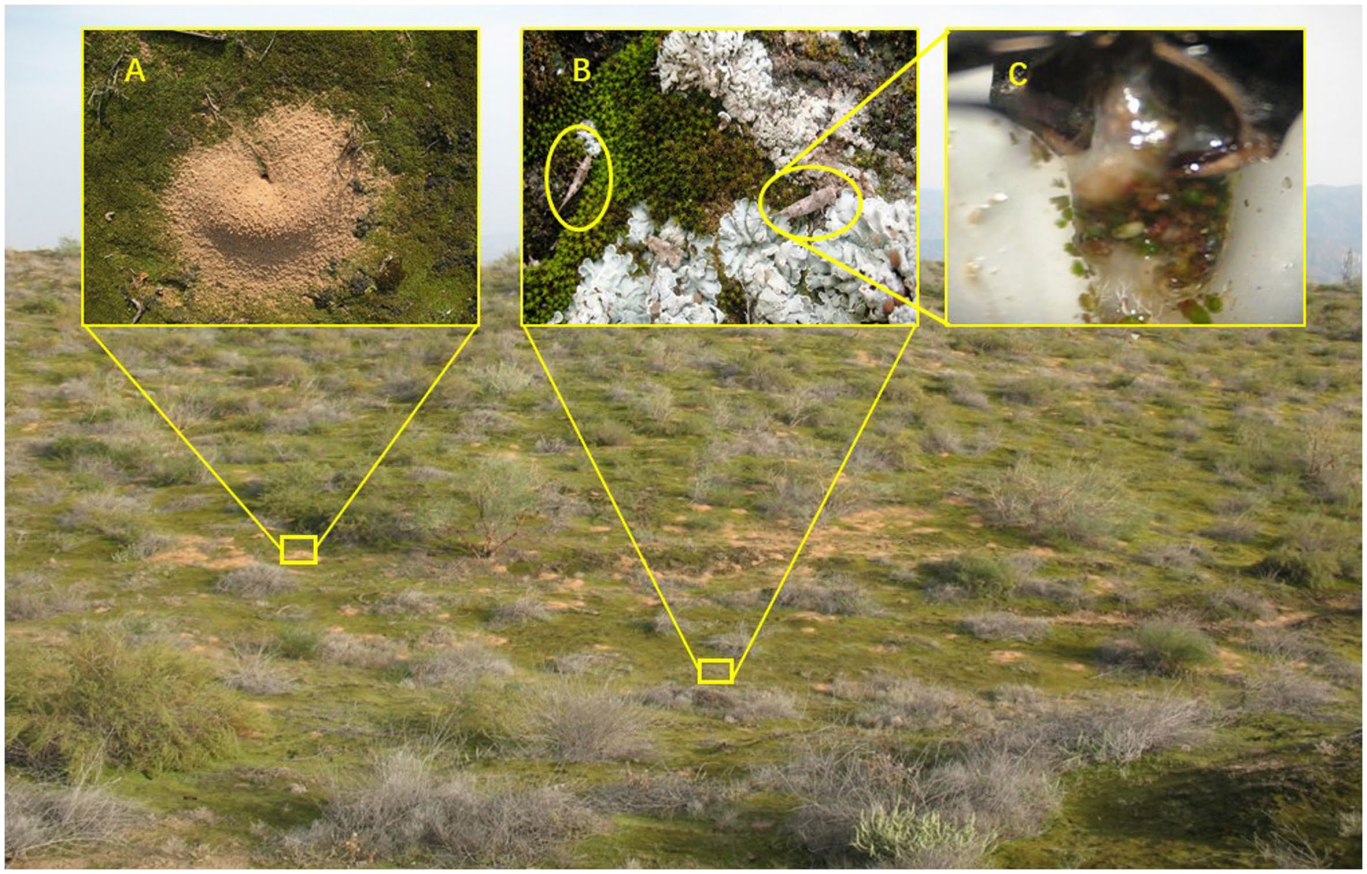
Biocrust provided both a novel habitat and food source for soil arthropod [**(A)** numerous ant nest occurred on biocrust covered dune surface; **(B)** *Haslundichilis* sp. were feeding on lichen and moss; and **(C)** moss and lichen were found in *Haslundichilis* sp. foregut].

Biocrusts also affect the seed germination, settlement, and survival of vascular plants by altering soil properties such as surface roughness, soil temperature, humidity, and nutrient content. Furthermore, biocrusts also affect the water content of shallow soils, thus increasing the species richness and biomass of herbs with shallow roots and reducing the coverage and biomass of woody plants with deep roots to ultimately increase the density of C_4_ plants (Li et al., 2010b, 2014b). The harsh environments of deserts and sand dunes threaten the survival of organisms. Biocrusts provide suitable habitats and food sources for soil arthropods (Figure 3). An increase in the biocrust cover on the sand surface was found to increase insect diversity (Li et al., 2011) and effectively protect an ant nest from damage by sand burial (Li et al., 2011, 2014d; Chen and Li, 2012). Further, biocrusts increase soil microbial richness and biomass (Liu et al., 2013; Yang et al., 2018). It should be noted that bacteria, fungi, and other microorganisms are fed upon by herbivorous and carnivorous-omnivorous nematodes (Zhi et al., 2009; Liu et al., 2011). The nematodes *Tylenchidae* and *Bursaphelenchus* directly feed on cyanobacteria and may also consume mosses and green algae (Zhi et al., 2009). An increase in nematode richness increases the richness of omnivorous-carnivorous organisms (Zhang et al., 2010; Guan et al., 2018). *Tenebrionidae* insects feed on mosses while *Microcoryphia* feed on lichens (Li et al., 2008b). These results demonstrated that biocrusts not only provide habitats for small soil animals but also directly participate in the composition of food chains in desert ecosystems.

In addition, small soil organisms such as bacteria on the epidermis of soil nematodes can affect biocrusts; specifically, certain bacteria can be excreted through the digestive system of nematodes, thus promoting the reproduction and colonisation of bacteria and indirectly promoting the colonisation of biocrusts (Zhi et al., 2009). Nest construction by ant *Formica cunicularia* Latr. can result in channels in the soil to consequently increase soil porosity and weaken rainfall interception by biocrusts (Li et al., 2011; Chen and Li, 2012).

## Biocrust Responses to Disturbances

Biocrust organisms are sensitive to erosion, sand burial, fire, grasing, and trampling due to their short stature and inhabiting shallow depths of soils. Wind erosion can cause direct mechanical damage to biocrust organisms, accelerate water loss, and inhibit photosynthesis, respiratory physiological activity, biomass accumulation, growth, and asexual reproduction of the biocrust (Jia et al., 2012). Sand burial is a physical stress which causes the mechanical compression of biocrusts (Jia et al., 2008) and reduces the availability of light and moisture (including dew) in crustal habitats (Rao et al., 2012; Jia et al., 2014). The effects of sand burial on biocrusts vary with the thickness and timing of the burial as well as the crust type. Shallow sand burial promotes biocrust growth, while thicker sand burial reduces PSII photochemical efficiency, chlorophyll a, and extracellular polysaccharide content of biocrusts. Long-term deep sand burial leads to the death of biocrust cryptogams (Wang et al., 2007). *Microcoleus vaginatus* Gom. can tolerate less than 1cm of sand burial by growth moving upwards (Rao et al., 2012). Sand burial thicknesses tolerated by mosses and lichens are greater than those tolerated by algae (Jia et al., 2008). Specifically, moss- and lichen-crust can tolerate burial depths ranging between 1 and 4 mm by reducing respiratory carbon losses and upward growth (Zhao et al., 2017). Sand burial is also expected to modify the species compositions of fungal communities (Grishkan et al., 2015) and the greenhouse gas fluxes of biocrust-covered soils (Jia et al., 2008).

The probability of fire occurrence in the desert regions of China is small because fires are controlled and prevented through management activities. However, occasional fires can significantly alter the compositions of crustal species, increase the coverage of cyanobacteria, and reduce the coverage of lichens and mosses (Li et al., 2016a). Additionally, fires can enhance the water repellency of moss-crusts (Wu and Liu, 2008) and inhibit the nitrogen fixation of *Collema tenax* (Sw.) Ach.em.Degel (Guo et al., 2016). Trampling has been shown to decrease the species richness, coverage, and surface stability of biocrusts (Liu et al., 2009; Wang et al., 2009b). Moreover, it can reduce the soil microbial biomass (Yang et al., 2018). However, the late-successional crusts have a higher tolerance to trampling disturbance compared to early-successional crusts (Wu et al., 2020). Further, damage to biocrusts can increase the likelihood of invasion by exotic species (Song et al., 2017a,b), which is likely to alter the multifunctionality of desert ecosystems (Li et al., 2013a).

## Applications for Land Degradation Control

The formation and passive restoration of biocrust under natural conditions occurs over a period of several decades (Zhao et al., 2011). The breeding of cyanobacteria, lichen, and moss can accelerate the formation of artificial biocrust and is suggested as an effective strategy for land degradation control (Xiao et al., 2015; Zhou et al., 2020).

Dominant cyanobacteria in biocrust such as *Microcoleus vaginatus* Gom. and *Scytonema javanicum* Born et Flah have been successfully isolated, cultivated, and employed as effective bio-materials to fix mobile dunes and prevent grasslands from sand burial in the Hobq Desert (Chen et al., 2006b; Wang et al., 2008b; Lan et al., 2014b). In this regard, physiological characteristics of the artificial cyanobacterial crust (Bu et al., 2014), its tolerance to stress (Chen et al., 2013), field soil moisture, temperature, light, and nutrient supply (Chen et al., 2006b), and its distribution on sand dunes (Li et al., 2013b) were determined. Specifically, these studies elucidated the appropriate range of light, temperature, and nutrient conditions, thus allowing establishment of the factory production process and development of the sand surface inoculation technology (Li et al., 2016a; Zhao et al., 2021).

Three common cyanobacteria (*Nostoc* sp., *Phormidium* sp., and *Scytonema arcangeli* Bornet ex Flahault) were isolated from a local biocrust in the Tengger Desert and subsequently cultured (Li et al., 2016a). Furthermore, the cyanobacteria were inoculated in the sands with a sand-fixing agent and a strong water-absorbent polymer. The hardness of the dune surface soil was significantly enhanced after an inoculation period of 1year. Further, the carbohydrate content, biomass, microbial biomass, soil respiration, carbon fixation, and effective quantum yield of the newly formed biocrust were 50–100% those of a natural biocrust (developed over a duration of 20years; Park et al., 2017). In addition, asexual reproduction of buds, stems, and leaves of certain mosses indicated the feasibility of cultivation of artificial moss-crusts (Xu et al., 2008; Bu et al., 2015c, 2018). Further, these results determined the optimum cultivation temperatures, humidity levels, nutrient solutions, nutrient concentrations, and substrate and field inoculation methods for *Tortula desertorum* Broth. in the Gurbantunggut Desert (Xu et al., 2008), *Bryum argenteum* Hedw. in the Tengger Desert and the Mu Us Desert Sandy land (Bu et al., 2015c; Li et al., 2016a), and *Didymodon vinealis* (Brid.) Zand in the Loess Plateau (Bu et al., 2017). In general, cyanobacteria can be successfully inoculated at a large area, while moss or lichen inoculation on large areas still faces many difficulties, and further research is needed on how inoculation affects vegetation diversity and structure and ecological processes (Zhou et al., 2020).

**Figure 4:**
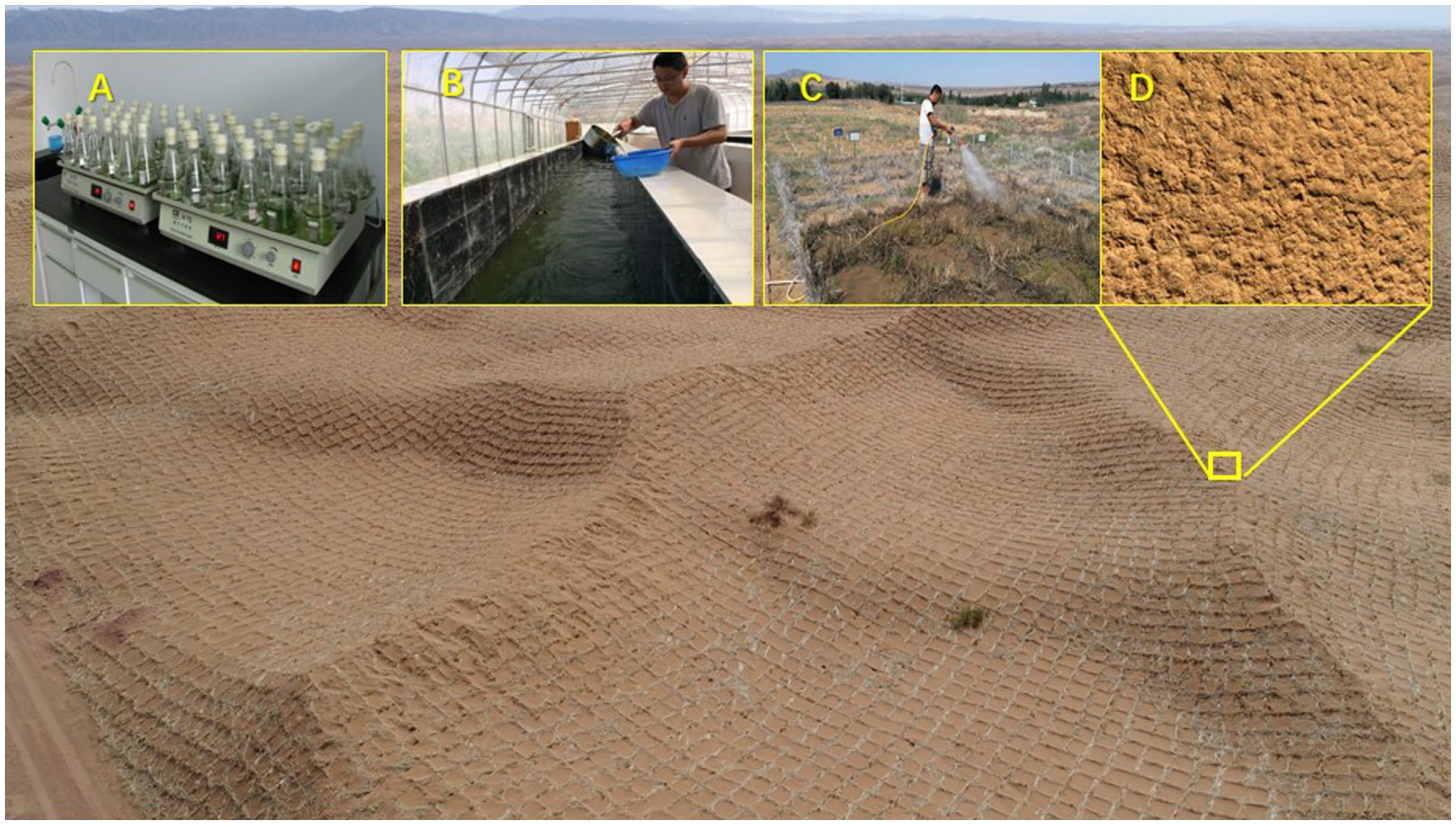
Cultivated non-vascular plants were employed to form biocrusts on dune surface provisionally fixed by the sand barrier using straw checkerboard [**(A)** strain isolation and purification; **(B)** industrial scaled-up cultivation; **(C)** field spray-inoculation; and **(D)** cyanobacteria dominated crust after 1year in the southeastern edge of the Tengger Desert].

However, artificial biocrusts can stabilise dunes and prevent sand burial by shortening the sand fixation time and improving the efficiency of sand fixation. Subsequently, a comprehensive approach based on these results has been suggested for land degradation control (Figure 4). This approach combines traditional revegetation techniques (e.g., establishing straw-checkboard sand barriers and planting xerophytic shrubs) with spraying artificial cyanobacteria or moss fixed solution (Li et al., 2016a), thus restoring land degradation in the arid and semiarid regions of China (Zhao et al., 2019).

## Conclusion

As can be seen from the above research progress, biocrust is good indicator of desert ecosystem health and sustainable development, as well as bio-materials with great potentiality for restoration of land degradation since biocrust prevents soil erosion and facilitates the establishment of plant and soil biome, as well as maintains water balance. Furthermore, biocrusts can rapidly cover on sand dunes by inoculating and cultivating related nonvascular species and their high tolerate to harsh conditions, including exposure to intense UV radiation, drought stress, and various biotic and abiotic disturbances. These findings well explored biocrust roles in soil ecological, hydrological, landscape, and biogeochemical processes, as well as in desert ecosystem self-organisation, well supplementing our knowledge gap on biocrust in temperate deserts. The research progresses during two decades since 2000 were also reflected in research scales, namely, evolving from the local to the regional to the landscape scale. Additionally, the methods and frameworks have shifted from traditional field observations and control experiments to the utilisation of molecular biology to explore underlying mechanisms, performing large-scale model simulations, and conducting multidisciplinary studies. Moreover, the research focuses have shifted from understanding the spatiotemporal distributions, compositions, structures, and functions of biocrusts to elucidating multi-scale ecosystem and landscape level processes and response mechanisms undergoing climate change. These include understanding the impacts of biocrusts on ecological restoration, important ecological processes such as C and N cycling in drylands, interaction between soil biomes, and maintenance of biodiversity and multifunctionality.

However, understanding on the underlying mutual feedback mechanisms of biocrust in ecological, hydrological, and biogeochemical processes is limited. Especially, we have limited understanding of the effects of global climate change on the ecosystem services of biocrusts, such as reducing the risk of biological invasions, dust emission of sand-dust storms and snowmelt, conserving biodiversity, maintaining water balance in global drylands, furthermore, clarifying the countermeasures to maintain its ecosystem services, etc. In addition, we still do not know whether the microorganisms in the biocrust pose a potential hazard to human health after it is broken. A largely ignored, but potentially important human exposure route for cyanotoxins in desert environments is through the inhalation of desert crusts during dust storms and anthropogenic activity. Future work in this field should include the characterisation of toxins produced in desert regions as well as the presence of toxins in clinical and environmental materials.

Finally, the species selection and inoculation techniques of artificial biocrust, including using net-work structured nanocomposite with high water-retention ability, viscosity, and biosafety as novel material for colonisation and development of artificial cyanobacteria, lichen, and moss on the sand surface in desertified grassland, and employment as a potential biofertilizer for soil reclamation, especially in terms of desertified land management, as well as other potential valuable bioresource such as pharmaceutical, animal feed, food (artificial cultivation of *Nostoc commune* and *Nostoc flagelliforme* as Chinese traditional food material), and fuel production should be the focus of future studies, because biocrust microalgae consist of a variety of components including carbohydrates, proteins, pigments, and lipids.

## Author Contributions

XL, RH, HT, RL, and NS contributed to manuscript design, analysis, and drafting. All authors contributed to the article and approved the submitted version.

## Funding

This research was supported by the National Natural Science Foundation of China (grant numbers 41621001 and 32061123006).

## Conflict of Interest

The authors declare that the research was conducted in the absence of any commercial or financial relationships that could be construed as a potential conflict of interest.

## Publisher’s Note

All claims expressed in this article are solely those of the authors and do not necessarily represent those of their affiliated organizations, or those of the publisher, the editors and the reviewers. Any product that may be evaluated in this article, or claim that may be made by its manufacturer, is not guaranteed or endorsed by the publisher.

## References

[ref1] Belnap J. (2003). The world at your feet: desert biological soil crusts. Front. Ecol. Environ. 1, 181–189. doi: 10.1890/1540-9295(2003)001[0181:TWAYFD]2.0.CO;2

[ref2] Belnap J. Lange O. L. (eds.) (2003). Biological Soil Crusts: Structure, Function, and Management. New York: Springer.

[ref3] Bu C. F. Li R. X. Wang C. Bowker M. A. (2018). Successful field cultivation of moss biocrusts on disturbed soil surfaces in the short term. Plant Soil 429, 227–240. doi: 10.1007/s11104-017-3453-0

[ref4] Bu C. F. Wang C. Yang Y. S. (2017). Physiological responses of artificial moss biocrusts to dehydration-rehydration process and heat stress on the loess plateau, China. J. Arid. Land 9, 419–431. doi: 10.1007/s40333-017-0057-8

[ref5] Bu C. F. Wu S. F. Han F. P. Yang Y. S. Meng J. (2015a). The combined effects of moss-dominated biocrusts and vegetation on erosion and soil moisture and implications for disturbance on the loess plateau, China. PLoS One 10:e0127394. doi: 10.1371/journal.pone.0127394, PMID: 25993431 PMC4439065

[ref6] Bu C. F. Wu S. F. Xie Y. S. Zhang X. (2013). The study of biological soil crusts: hotspots and prospects. Clean Soil Air Water 41, 899–906. doi: 10.1002/clen.201100675

[ref7] Bu C. Wu S. Yang Y. Zheng M. (2014). Identification of factors influencing the restoration of cyanobacteria-dominated biological soil crusts. PLoS One 9:e90049. doi: 10.1371/journal.pone.0090049, PMID: 24625498 PMC3953112

[ref8] Bu C. F. Zhang K. K. Zhang C. Y. Wu S. F. (2015c). Key factors influencing rapid development of potentially dune-stabilizing moss-dominated crusts. PLoS One 10:e0134447. doi: 10.1371/journal.pone.0134447, PMID: 26230324 PMC4521833

[ref9] Bu C. F. Zhao Y. Robert L. H. Zhao C. L. Yang Y. S. Zhang P.. (2015b). Wind erosion prevention characteristics and key influencing factors of bryophytic soil crusts. Plant Soil 397, 163–174. doi: 10.1007/s11104-015-2609-z

[ref10] Chen L. Z. Deng S. Q. De Philippis R. Tian W. Wu H. Wang J. (2013). UV-B resistance as a criterion for the selection of desert microalgae to be utilized for inoculating desert soils. J. Appl. Phycol. 25, 1009–1015. doi: 10.1007/s10811-012-9906-1

[ref11] Chen X. H. Duan Z. H. (2015). Impacts of soil crusts on soil physicochemical characteristics in different rainfall zones of the arid and semi-arid desert regions of northern China. Environ. Earth Sci. 73, 3335–3347. doi: 10.1007/s12665-014-3622-x

[ref12] Chen Y. W. Li X. R. (2012). Spatiotemporal distribution of nests and influence of ant (*Formica cunicularia* Lat.) activity on soil property and seed bank after revegetation in the Tengger Desert. Arid Land Res. Manag. 26, 365–378. doi: 10.1080/15324982.2012.694393

[ref13] Chen L. Z. Li D. H. Liu Y. D. (2003). Salt tolerance of Microcoleus vaginatus Gom., a cyanobacterium isolated from desert algal crust, was enhanced by exogenous carbohydrates. J. Arid Environ. 55, 645–656. doi: 10.1016/S0140-1963(02)00292-6

[ref14] Chen L. Z. Li D. H. Song L. R. Hu C. X. Wang G. H. Liu Y. D. (2006a). Effects of salt stress on carbohydrate metabolism in desert soil alga Microcoleus vaginatus Gom. J. Integr. Plant Biol. 48, 914–919. doi: 10.1111/j.1744-7909.2006.00291.x

[ref15] Chen L. Z. Wang G. H. Hong S. (2009). UV-B-induced oxidative damage and protective role of exopolysaccharides in desert cyanobacterium Microcoleus vaginatus. J. Integr. Plant Biol. 51, 194–200. doi: 10.1111/j.1744-7909.2008.00784.x, PMID: 19200158

[ref16] Chen L. Z. Xie Z. M. Bi Y. H. Wang G. H. Deng S. Q. Liu Y. D. (2012). The combined effects of UV-B radiation and herbicides on photosynthesis, antioxidant enzymes and DNA damage in two bloom-forming cyanobacteria. Ecotoxicol. Environ. Saf. 80, 224–230. doi: 10.1016/j.ecoenv.2012.03.007, PMID: 22464588

[ref17] Chen L. Z. Xie Z. Hu C. Li D. Wang G. Liu Y. (2006b). Man-made desert algal crusts as affected by environmental factors in inner Mongolia, China. J. Arid Environ. 67, 521–527. doi: 10.1016/j.jaridenv.2006.02.018

[ref18] Chen R. Y. Zhang Y. M. Li Y. Wei W. Zhang J. Wu N. (2008). The variation of morphological features and mineralogical components of biological soil crusts in the gurbantunggut desert of northwestern China. Environ. Geol. 57, 1135–1143. doi: 10.1007/s00254-008-1410-1

[ref19] Eldridge D. J. Greene R. S. B. (1994). Microbiotic soil crusts: a review of their roles in soil and ecological processes in the rangelands of Australia. Aust. J. Soil Res. 32, 389–415. doi: 10.1071/SR9940389

[ref20] Gao L. Bowker M. A. Sun H. Zhao J. Zhao Y. G. (2020a). Linkages between biocrust development and water erosion and implications for erosion model implementation. Geoderma 357:113973. doi: 10.1016/j.geoderma.2019.113973

[ref21] Gao L. Bowker M. A. Xu M. Sun H. Tuo D. Zhao Y. (2017a). Biological soil crusts decrease erodibility by modifying inherent soil properties on the loess plateau, China. Soil Biol. Biochem. 105, 49–58. doi: 10.1016/j.soilbio.2016.11.009

[ref22] Gao Y. H. Li X. R. Liu L. C. Jia R. L. Yang H. T. Li G.. (2012). Seasonal variation of carbon exchange from a revegetation area in a Chinese desert. Agric. For. Meteorol. 156, 134–142. doi: 10.1016/j.agrformet.2012.01.007

[ref23] Gao B. Li X. S. Zhang D. Y. Liang Y. Yang H. (2017b). Desiccation tolerance in bryophytes: the dehydration and rehydration transcriptomes in the desiccation-tolerant bryophyte *Bryum argenteum*. Sci. Rep. 7:7571. doi: 10.1038/s41598-017-07297-3, PMID: 28790328 PMC5548717

[ref24] Gao L. Sun H. Xu M. X. Zhao Y. G. (2020b). Biocrusts resist runoff erosion through direct physical protection and indirect modification of soil properties. J. Soils Sediments 20, 133–142. doi: 10.1007/s11368-019-02372-w

[ref25] Gao S. Q. Ye X. H. Chu Y. Dong M. (2010). Effects of biological soil crusts on profile distribution of soil water, organic carbon and total nitrogen in mu us Sandland, China. J. Plant Ecol. 3, 279–284. doi: 10.1093/jpe/rtq015

[ref26] Ge H. M. Zhang J. Zhou X. P. Xia L. Hu C. X. (2014a). Effects of light intensity on component and topographical structure of extracellular polysaccharide from the cyanobacteria *Nostoc* sp. J. Microbiol. 52, 179–183. doi: 10.1007/s12275-014-2720-5, PMID: 24500483

[ref27] Ge H. Zhang J. Zhou X. Xia L. Hu C. (2014b). Effects of light intensity on components and topographical structures of extracellular polymeric substances from *Microcoleus vaginatus* (Cyanophyceae). Phycologia 53, 167–173. doi: 10.2216/13-163.1

[ref28] Grishkan I. Jia R. L. Kidron G. J. Li X. R. (2015). Cultivable microfungal communities inhabiting biological soil crusts in the tengger desert, China. Pedosphere 25, 351–363. doi: 10.1016/S1002-0160(15)30003-5

[ref29] Guan P. T. Zhang X. K. Yu J. Sheng Y. Li Q. (2018). Soil microbial food web channels associated with biological soil crusts in desertification restoration: the carbon flow from microbes to nematodes. Soil Biol. Biochem. 116, 82–90. doi: 10.1016/j.soilbio.2017.10.003

[ref30] Guan C. Zhang P. Zhao C. M. Li X. R. (2021). Effects of warming and rainfall pulses on soil respiration in a biological soil crust-dominated desert ecosystem. Geoderma 381:114683. doi: 10.1016/j.geoderma.2020.114683

[ref31] Guo C. J. Chen L. Xiao B. (2016). Soil water repellency of moss-dominated biological soil crusts and their response to fire duration on the loess plateau of China. J. Shenyang Agricul. Univ. 47, 212–217.

[ref32] Guo Y. R. Zhao H. L. Zuo X. A. Drake S. Zhao X. Y. (2008). Biological soil crust development and its topsoil properties in the process of dune stabilization, inner Mongolia, China. Environ. Geol. 54, 653–662. doi: 10.1007/s00254-007-1130-y

[ref33] Hong Y. Li Y. Y. Li S. H. (1992). Preliminary study on the blue-green algae community of arid soil in Qaidam basin. Acta Bot. Sin. 34, l6l–l168l.

[ref34] Hu C. X. Liu Y. D. (2003). Primary succession of algal community structure in desert soil. Acta Bot. Sin. 45, 917–924.

[ref35] Hu C. X. Liu Y. D. Zhang D. L. (2002). Cementing mechanism of algal crusts from desert area. Chin. Sci. Bull. 47, 1361–1368. doi: 10.1360/02tb9301

[ref36] Hu R. Wang X. P. Pan Y. X. Zhang Y. F. Zhang H. (2014). The response mechanisms of soil N mineralization under biological soil crusts to temperature and moisture in temperate desert regions. Eur. J. Soil Biol. 62, 66–73. doi: 10.1016/j.ejsobi.2014.02.008

[ref37] Hu R. Wang X. P. Pan Y. X. Zhang Y. F. Zhang H. (2015). Seasonal variation of net N mineralization under different biological soil crusts in tengger desert, North China. Catena 127, 9–16. doi: 10.1016/j.catena.2014.12.012

[ref38] Hu R. Wang X. P. Zhang Y. F. Shi W. Jin Y. X. Chen N. (2016). Insight into the influence of sand-stabilizing shrubs on soil enzyme activity in a temperate desert. Catena 137, 526–535. doi: 10.1016/j.catena.2015.10.022

[ref39] Hu C. X. Zhang D. L. Huang Z. B. Liu Y. D. (2013). The vertical microdistribution of cyanobacteria and green algae within desert crusts and the development of the algal crusts. Plant Soil 257, 97–111. doi: 10.1023/A:1026253307432

[ref40] Hu Y. G. Zhang Z. S. Huang L. Qi Q. Liu L. C. Zhao Y.. (2019). Shifts in soil microbial community functional gene structure across a 61-year desert revegetation chronosequence. Geoderma 347, 126–134. doi: 10.1016/j.geoderma.2019.03.046

[ref41] Hu C. X. Zhang D. L. Liu Y. D. (2004). Research progress on algae of the microbial crusts in arid and semiarid regions. Prog. Nat. Sci. 14, 289–295. doi: 10.1080/10020070412331343501

[ref42] Huang L. Zhang Z. S. Li X. R. (2014a). Carbon fixation and its influence factors of biological soil crusts in a revegetated area of the tengger desert, northern China. J. Arid. Land 6, 725–734. doi: 10.1007/s40333-014-0027-3

[ref43] Huang L. Zhang Z. S. Li X. R. (2014b). Soil CO_2_ concentration in biological soil crust and its factors revegetation area of the tengger desert, northern China. Environ. Earth Sci. 72, 767–777. doi: 10.1007/s12665-013-3000-0

[ref44] Hui R. Li X. R. Chen C. Y. Zhao X. Jia R. L. Liu L. C.. (2013). Responses of photosynthetic properties and chloroplast ultrastructure of *Bryum argenteum* from a desert biological soil crust to elevated ultraviolet-B radiation. Physiol. Plant. 147, 489–501. doi: 10.1111/j.1399-3054.2012.01679.x, PMID: 22901234

[ref45] Hui R. Li X. R. Jia R. L. Liu L. C. Zhao R. M. Zhao X.. (2014). Photosynthesis of two moss crusts from the tengger desert with contrasting sensitivity to supplementary UV-B radiation. Photosynthetica 52, 36–49. doi: 10.1007/s11099-014-0003-3

[ref46] Hui R. Li X. R. Zhao R. M. Liu L. C. Gao Y. H. Wei Y. P. (2015). UV-B radiation suppresses chlorophyll fluorescence, photosynthetic pigment and antioxidant systems of two key species in soil crusts from the tengger desert, China. J. Arid Environ. 113, 6–15. doi: 10.1016/j.jaridenv.2014.08.007

[ref47] Hui R. Li X. R. Zhao R. M. Tan H. J. Jia R. L. (2021). Physiological response of moss/cyanobacteria crusts along a precipitation gradient from semi-arid to arid desert in China. Plant Soil. doi: 10.1007/s11104-021-05117-2

[ref48] Hui R. Zhao R. M. Liu L. C. Li G. Yang H. T. Gao Y. H.. (2016a). Modelling the influence of snowfall on cyanobacterial crusts in the gurbantunggut desert, northern China. Aust. J. Bot. 64, 476–483. doi: 10.1071/bt16008

[ref49] Hui R. Zhao R. M. Liu L. C. Zh R. Q. Li G. We Y. P. (2016b). Effects of UV-B, water deficit and their combination on *Bryum argenteum* plants. Russ. J. Plant Physiol. 63, 231–238. doi: 10.1134/S1021443716020084

[ref50] Hui R. Zhao R. M. Song G. Li Y. X. Zhao Y. Wang Y. L. (2018). Effects of enhanced ultraviolet-B radiation, water deficit, and their combination on UV-absorbing compounds and osmotic adjustment substances in two different moss species. Environ. Sci. Pollut. Res. 25, 14953–14963. doi: 10.1007/s11356-018-1689-8, PMID: 29549614

[ref51] Jia R. L. Li X. R. Liu L. C. Gao Y. H. (2014). Effects of sand burial on dew deposition on moss soil crust in a revegetated area of the tengger desert, northern China. J. Hydrol. 519, 2341–2349. doi: 10.1016/j.jhydrol.2014.10.031

[ref52] Jia R. L. Li X. R. Liu L. C. Gao Y. H. Li X. J. (2008). Responses of biological soil crusts to sand burial in a revegetated area of the tengger desert, northern China. Soil Biol. Biochem. 40, 2827–2834. doi: 10.1016/j.soilbio.2008.07.029

[ref53] Jia R. L. Li X. R. Liu L. C. Gao Y. H. Zhang X. T. (2012). Differential wind tolerance of soil crust mosses explains their micro-distribution in nature. Soil Biol. Biochem. 45, 31–39. doi: 10.1016/j.soilbio.2011.09.021

[ref54] Lan S. B. Ouyang H. L. Wu L. Zhang D. L. Hu C. X. (2017). Biological soil crust community types differ in photosynthetic pigment composition, fluorescence and carbon fixation in shapotou region of China. Appl. Soil Ecol. 111, 9–16. doi: 10.1016/j.apsoil.2016.11.009

[ref55] Lan S. Wu L. Zhang D. Hu C. (2012). Successional stages of biological soil crusts and their microstructure variability in Shapotou region (China). Environ. Earth Sci. 65, 77–88. doi: 10.1007/s12665-011-1066-0

[ref56] Lan S. B. Wu L. Zhang D. L. Hu C. X. (2014a). Desiccation provides photosynthetic protection for crust cyanobacteria *Microcoleus vaginatus* from high temperature. Physiol. Plant. 152, 345–354. doi: 10.1111/ppl.12176, PMID: 24611508

[ref57] Lan S. B. Wu L. Zhang D. L. Hu C. X. (2015). Analysis of environmental factors determining development and succession in biological soil crusts. Sci. Total Environ. 538, 492–499. doi: 10.1016/j.scitotenv.2015.08.066, PMID: 26318686

[ref58] Lan S. Wu L. Zhang D. Hu C. Liu Y. (2010). Effects of drought and salt stresses on man-made cyanobacterial crusts. Eur. J. Soil Biol. 46, 381–386. doi: 10.1016/j.ejsobi.2010.08.002

[ref59] Lan S. B. Zhang Q. Y. Wu L. Liu Y. Zhang D. Hu C. (2014b). Artificially accelerating the reversal of desertification: cyanobacterial inoculation facilitates the succession of vegetation communities. Environ. Sci. Technol. 48, 307–315. doi: 10.1021/es403785j, PMID: 24303976

[ref60] Li X. R. (2012). Study on Eco-Hydrology of Desert Biological Soil Crusts. Beijing: High Education Press.

[ref61] Li S. L. Bowker M. A. Xiao B. (2021b). Biocrusts enhance non-rainfall water deposition and alter its distribution in dryland soils. J. Hydrol. 595:126050. doi: 10.1016/j.jhydrol.2021.126050

[ref62] Li X. R. Chen Y. W. Jia R. L. (2008b). Biological soil crusts: a significant food source for inserts in the arid desert ecosystems. J. Desert Res. 28, 245–248.

[ref63] Li X. R. Chen Y. W. Yang L. W. (2004b). Cryptogam diversity and formation of soil crusts in temperate desert. Ann. Arid Zone 43, 335–353.

[ref64] Li X. R. Gao Y. H. Su J. Q. Jia R. L. Zhang Z. S. (2014d). Ants mediate soil water in arid desert ecosystems: mitigating rainfall interception induced by biological soil crusts? Appl. Soil Ecol. 78, 57–64. doi: 10.1016/j.apsoil.2014.02.009

[ref65] Li X. R. He M. Z. Duan Z. H. Xiao H. L. Jia X. H. (2007b). Recovery of topsoil physicochemical properties in revegetated sites in the sand-burial ecosystems of the tengger desert, northern China. Geomorphology 88, 254–265. doi: 10.1016/j.geomorph.2006.11.009

[ref66] Li X. R. He M. Z. Zerbe S. Li X. J. Liu L. C. (2010a). Micro-geomorphology determines community structure of biological soil crusts at small scales. Earth Surf. Process. Landf. 35, 932–940. doi: 10.1002/esp.1963

[ref67] Li X. R. Hui R. Zhang P. Song N. P. (2021a). Divergent responses of moss- and lichen-dominated biocrusts to warming and increased drought in arid desert regions. Agric. For. Meteorol. 303:108387. doi: 10.1016/j.agrformet.2021.108387

[ref68] Li X. R. Hui R. Zhao Y. (2016a). Eco-Physiology of Biological Soil Crusts in Desert Regions of China. Beijing: High Education Press.

[ref69] Li X. R. Jia R. L. Chen Y. W. Huaang L. (2011). Association of ant nests with successional stages of biological soil crusts in the tengger desert, northern China. Appl. Soil Ecol. 47, 59–66. doi: 10.1016/j.apsoil.2010.10.010

[ref70] Li X. R. Jia X. H. Long L. Q. Zerb S. (2005). Effects of biological soil crusts on seed bank, germination and establishment of two annual plant species in the tengger desert (N China). Plant Soil 277, 375–385. doi: 10.1007/s11104-005-8162-4

[ref71] Li X. R. Jia R. L. Zhang Z. S. Zhang P. Hui R. (2018). Hydrological response of biological soil crusts to global warming: a ten-year simulative study. Glob. Chang. Biol. 24, 4960–4971. doi: 10.1111/gcb.14378, PMID: 29957890

[ref72] Li X. R. Kong D. S. Tan H. J. Wang X. P. (2007a). Changes in soil and in vegetation following stabilization of dune in southeastern fringe of the tengger desert, China. Plant Soil 300, 221–231. doi: 10.1007/s11104-007-9407-1

[ref73] Li J. H. Li X. R. Chen C. Y. (2014b). Degradation and reorganization of thylakoid proteins complexes are involved in the rapid photosynthetic changes of desert moss *Bryum argenteum* in response to dehydration and rehydration. Bryologist 117, 110–118. doi: 10.1639/0007-2745-117.2.110

[ref74] Li X. J. Li X. R. Song W. M. Gao Y. P. Zhang J. G. Jia R. L. (2008a). Effects of crust and shrub patches on runoff, sedimentation, and related nutrient (C, N) redistribution in the desertified steppe zone of the tengger desert, northern China. Geomorphology 96, 221–232. doi: 10.1016/j.geomorph.2007.08.006

[ref75] Li X. J. Li X. R. Wang X. P. Yang H. T. (2016b). Changes in soil organic carbon fractions after afforestation with xerophytic shrubs in the tengger desert, northern China. Eur. J. Soil Sci. 67, 184–195. doi: 10.1111/ejss.12315

[ref76] Li J. H. Li X. R. Zhang P. (2014a). Micro-morphology, ultrastructure and chemical composition changes of *Bryum argenteum* from a desert biological soil crust following one-year desiccation. Bryologist 117, 232–240. doi: 10.1639/0007-2745-117.3.232

[ref77] Li H. Rao B. Wang G. Shen S. Li D. Hu C.. (2013b). Spatial heterogeneity of cyanobacteria-inoculated sand dunes significantly influences artificial biological soil crusts in the hopq desert (China). Environ. Earth Sci. 71, 245–253. doi: 10.1007/s12665-013-2428-6

[ref78] Li X. R. Song G. Hui R. Wang Z. R. (2017). Precipitation and topsoil attributes determine the species diversity and distribution patterns of crustal communities in desert ecosystems. Plant Soil 420, 163–175. doi: 10.1007/s11104-017-3385-8

[ref79] Li X. R. Tian F. Jia R. L. Zhang Z. S. Liu L. C. (2010b). Do biological soil crusts determine vegetation changes in sandy deserts? Implications for managing artificial vegetation. Hydrol. Process. 24, 3621–3630. doi: 10.1002/hyp.7791

[ref80] Li X. R. Wang X. P. Li T. Zhang J. G. (2002). Microbiotic crust and its effect on vegetation and habitat on artificially stabilized desert dunes in tengger desert, North China. Biol. Fertil. Soils 35, 147–154. doi: 10.1007/s00374-002-0453-9

[ref81] Li T. Xiao H. L. Li X. R. (2001). Modeling the effects of crust on rain infiltration in vegetated sand dunes in arid desert. Arid Land Res. Manag. 15, 41–48. doi: 10.1080/153249801300000806

[ref82] Li S. L. Xiao B. Sun F. H. Kidron G. J. (2021c). Moss-dominated biocrusts enhance water vapor sorption capacity of surface soil and increase non-rainfall water deposition in drylands. Geoderma 388:114930. doi: 10.1016/j.geoderma.2021.114930

[ref83] Li X. R. Xiao H. L. Zhang J. G. Wang X. P. (2004a). Long-term ecosystem effects of sand-binding vegetation in the tengger desert, northern China. Restor. Ecol. 12, 376–390. doi: 10.1111/j.1061-2971.2004.00313.x

[ref84] Li X. R. Zhang Z. S. Huang L. Liu L. C. Wang X. P. (2009). The ecohydrology of the soil-vegetation system restoration in arid zones: a review. Sci. Cold Arid Regions 1, 199–206.

[ref85] Li X. R. Zhang Z. S. Huang L. Wang X. P. (2013a). Review of the ecohydrological processes and feedback mechanisms controlling sand-binding vegetation systems in sandy desert regions of China. Chin. Sci. Bull. 58, 1483–1496. doi: 10.1007/s11434-012-5662-5

[ref86] Li X. R. Zhang P. Su Y. G. Jia R. L. (2012). Carbon fixation by biological soil crusts following revegetation of sand dunes in arid desert regions of China: a four-year field study. Catena 97, 119–126. doi: 10.1016/j.catena.2012.05.009

[ref87] Li X. R. Zhang Z. S. Tan H. J. Gao Y. H. Liu L. C. Wang X. P. (2014c). Ecological restoration and recovery in the wind-blown sand hazard areas of northern China: relationship between soil water and carrying capacity for vegetation in the tengger desert. Sci. China Life Sci. 57, 539–548. doi: 10.1007/s11427-014-4633-2, PMID: 24699917

[ref88] Li X. R. Zhang J. G. Wang X. P. Liu L. C. (2000). Study on soil microbiotic crust and its influences on sand fixing vegetation in arid desert region. Acta Bot. Sin. 42, 965–970.

[ref89] Li X. R. Zhou H. Y. Wang X. P. Zhu Y. G. O’Conner P. J. (2003). The effects of sand stabilization and revegetation on cryptogam species diversity and soil fertility in tengger desert, northern China. Plant Soil 251, 237–245. doi: 10.1023/A:1023023702248

[ref90] Liu M. (2012). A research on desert lichen diversity in Shapotou region of the Tengger Desert, China. Master Dissertation. Taian: Shandong Agricultural University.

[ref92] Liu Y. R. Delgado-Baquerizo M. Trivedi P. He J. Z. Singh B. K. (2016b). Species identity of biocrust-forming lichens drives the response of soil nitrogen cycle to altered precipitation frequency and nitrogen amendment. Soil Biol. Biochem. 96, 128–136. doi: 10.1016/j.soilbio.2016.01.021

[ref93] Liu H. J. Han X. G. Li L. H. Huang J. H. Liu H. S. Li X. (2009). Grazing density effects on cover, species composition, and nitrogen fixation of biological soil crust in an Inner Mongolia steppe. Rangel. Ecol. Manag. 62, 321–327. doi: 10.2111/08-179.1

[ref94] Liu L. C. Li S. Z. Duan Z. H. Wang T. Zhang Z. S. Li X. R. (2006). Effects of microbiotic crusts on dew deposition in the restored vegetation area at shapotou, Northwest China. J. Hydrol. 328, 331–337. doi: 10.1016/j.jhydrol.2005.12.004

[ref95] Liu Y. M. Li X. R. Jia R. L. Huang L. Zhou Y. Y. Gao Y. H. (2011). Effects of biological soil crusts on soil nematode communities following dune stabilization in the tengger desert, northern China. Appl. Soil Ecol. 49, 118–124. doi: 10.1016/j.apsoil.2011.06.007

[ref96] Liu Y. M. Li X. R. Xing Z. S. Zhao X. Pan Y. X. (2013). Responses of soil microbial biomass and community composition to biological soil crusts in the revegetated areas of the tengger desert. Appl. Soil Ecol. 65, 52–59. doi: 10.1016/j.apsoil.2013.01.005

[ref97] Liu L. C. Liu Y. B. Hui R. Xie M. (2017). Recovery of microbial community structure of biological soil crusts in successional stages of Shapotou desert revegetation, Northwest China. Soil Biol. Biochem. 107, 125–128. doi: 10.1016/j.soilbio.2016.12.030

[ref98] Liu L. C. Song Y. X. Gao Y. H. Wang T. Li X. R. (2007). Effects of microbiotic crusts on evaporation from the revegetated area in a Chinese desert. Aust. J. Soil Res. 45, 422–427. doi: 10.1071/SR06175

[ref99] Liu Y. M. Yang H. Y. Li X. R. Xing Z. S. (2014). Effects of biological soil crusts on soil enzyme activities in revegetated areas of the tengger desert, China. Appl. Soil Ecol. 80, 6–14. doi: 10.1016/j.apsoil.2014.03.015

[ref100] Liu F. Zhang G. H. Sun L. Wang H. (2016a). Effects of biological soil crusts on soil detachment process by overland flow in the loess plateau of China. Earth Surf. Process. Landf. 41, 875–883. doi: 10.1002/esp.3870

[ref101] Liu Y. B. Zhao L. N. Wang Z. R. Liu L. C. Zhang P. Sun J. Y.. (2018). Changes in functional gene structure and metabolic potential of the microbial community in biological soil crusts along a revegetation chronosequence in the tengger desert. Soil Biol. Biochem. 120, 40–48. doi: 10.1016/j.soilbio.2018.08.012

[ref102] Ma Z. L. Helbling E. W. Li W. Villafane V. E. Gao K. (2012). Motility and photosynthetic responses of the green microalga *Tetraselmis subcordiformis* to visible and UV light levels. J. Appl. Phycol. 24, 1613–1621. doi: 10.1007/s10811-012-9822-4

[ref103] Niu J. Yang K. Tang Z. Wang Y. (2017). Relationships between soil crust development and soil properties in the desert region of North China. Sustainability 9:725. doi: 10.3390/su9050725

[ref104] Ouyang H. L. Hu C. X. (2017). Insight into climate change from the carbon exchange of biocrusts utilizing non-rainfall water. Sci. Rep. 7:2573. doi: 10.1038/s41598-017-02812-y, PMID: 28566698 PMC5451392

[ref105] Pan Z. Pitt W. G. Zhang Y. M. Wu N. Tao Y. Truscott T. (2016). The upside-down water collection system of *Syntrichia caninervis*. Nat. Plants 2:16076. doi: 10.1038/nplants.2016.76, PMID: 27302768

[ref106] Pan Y. X. Wang X. P. (2014). Effects of shrub species and microhabitats on dew formation in a revegetation-stabilized desert ecosystem in shapotou, northern China. J. Arid. Land 6, 389–399. doi: 10.1007/s40333-014-0008-6

[ref107] Pan Y. X. Wang X. P. Zhang Y. F. (2010). Dew formation characteristics in a revegetation-stabilized desert ecosystem in shapotou area, northern China. J. Hydrol. 387, 265–272. doi: 10.1016/j.jhydrol.2010.04.016

[ref108] Park C. H. Li X. R. Zhao Y. Jia R. L. Hur J.-S. (2017). Rapid development of cyanobacterial crust in the field for combating desertification. PLoS One 12:e0179903. doi: 10.1371/journal.pone.0189342, PMID: 28644849 PMC5482470

[ref109] Qi J. H. Liu Y. B. Wang Z. R. Zhao L. N. Zhang W. J. Wang Y. S.. (2021). Variations in microbial functional potential associated with phosphorus and sulfur cycling in biological soil crusts of different ages at the tengger desert, China. Appl. Soil Ecol. 165:104022. doi: 10.1016/j.apsoil.2021.104022

[ref110] Rao B. Q. Liu Y. D. Lan S. B. Wu P. P. Wang W. B. Li D. H. (2012). Effects of sand burial stress on the early developments of cyanobacterial crusts in the field. Eur. J. Soil Biol. 48, 48–55. doi: 10.1016/j.ejsobi.2011.07.009

[ref111] Rao B. Q. Liu Y. D. Wang W. B. Hu C. X. Li D. H. Lan S. B. (2009). Influence of dew on biomass and photosystem II activity of cyanobacterial crusts in the hopq desert, Northwest China. Soil Biol. Biochem. 41, 2387–2393. doi: 10.1016/j.soilbio.2009.06.005

[ref112] Reynaud P. A. Lumpkin T. A. (1988). Microalgae of the Lanzhou (China) cryptogamic crust. Arid Soil Res. Rehabil. 2, 145–155. doi: 10.1080/15324988809381169

[ref113] Rodriguez-Caballero E. Belnap J. Büdel B. Crutzen P. J. Andreae M. O. Pochl U.. (2018). Dryland photoautotrophic soil surface communities endangered by global change. Nat. Geosci. 11, 185–189. doi: 10.1038/s41561-018-0072-1

[ref114] Song G. Li X. R. Hui R. (2017a). Effect of biological soil crusts on seed germination and growth of an exotic and two native plant species in an arid ecosystem. PLoS One 12:e0185839. doi: 10.1371/journal.pone.0185839, PMID: 28977018 PMC5627943

[ref115] Song G. Li X. R. Hui R. (2017b). Biological soil crusts determine the germination and growth of two exotic plants. Ecol. Evol. 7, 9441–9450. doi: 10.1002/ece3.3477, PMID: 29187980 PMC5696392

[ref116] Su Y. G. Li X. R. Chen Y. W. Zhang Z. S. Lu Y. (2013b). Carbon fixation of cyanobacterial-algal crusts after desert fixation and its implication to soil organic carbon accumulation in desert. Land Degrad. Dev. 24, 342–349. doi: 10.1002/ldr.1131

[ref117] Su Y. G. Li X. R. Cheng Y. W. (2007). Effects of biological soil crusts on emergence of desert vascular plants in North China. Plant Ecol. 191, 11–19. doi: 10.1007/s11258-006-9210-8

[ref118] Su Y. G. Li X. R. Zheng J. G. Huang G. (2009). The effect of biological soil crusts of different successional stages and conditions on the germination of seeds of three desert plants. J. Arid Environ. 73, 931–936. doi: 10.1016/j.jaridenv.2009.04.010

[ref119] Su Y. G. Liu J. Zhang Y. M. Huang G. (2020). More drought leads to a greater significance of biocrusts to soil multifunctionality. Funct. Ecol. 35, 989–1000. doi: 10.1111/1365-2435.13761

[ref120] Su Y. G. Wu L. Zhou Z. B. Zhang Y. M. (2013a). Carbon flux in deserts depends on soil cover type: a case study in the gurbantunggute desert, North China. Soil Biol. Biochem. 58, 332–340. doi: 10.1016/j.soilbio.2012.12.006

[ref121] Su Y. G. Zhao X. Li A. X. Li X. R. Huang G. (2011). Nitrogen fixation in biological soil crusts from the tengger desert, northern China. Eur. J. Soil Biol. 47, 182–187. doi: 10.1016/j.ejsobi.2011.04.001

[ref122] Sun Y. L. Li X. Y. Xu H. Y. Yang Z. Tang J. (2008). Effect of soil crust on evaporation and dew deposition in mu us sandy land, China. Front. Environ. Sci. Eng. China 2, 480–486. doi: 10.1007/s11783-008-0034-8

[ref123] Tang D. Shi S. Li D. Hu C. Liu Y. (2007). Physiological and biochemical responses of *Scytonema javanicum* (cyanobacterium) to salt stress. J. Arid Environ. 71, 312–320. doi: 10.1016/j.jaridenv.2007.05.004

[ref124] Tao Y. Zhang Y. M. (2012). Effects of leaf hair points of a desert moss on water retention and dew formation: implications for desiccation tolerance. J. Plant Res. 125, 351–360. doi: 10.1007/s10265-011-0449-3, PMID: 22089730

[ref125] Wang J. Bao J. T. Su J. Q. Li X. R. Chen G. X. (2015). Impact of inorganic nitrogen additions on microbes in biological soil crusts. Soil Biol. Biochem. 88, 303–313. doi: 10.1016/j.soilbio.2015.06.004

[ref126] Wang G. H. Chen K. Chen L. Z. Hu C. Zhang D. Liu Y. (2008a). The involvement of the antioxidant system in protection of desert cyanobacterium *Nostoc* sp. against UV-B radiation and the effects of exogenous antioxidants. Ecotoxicol. Environ. Saf. 69, 150–157. doi: 10.1016/j.ecoenv.2006.03.014, PMID: 16759702

[ref127] Wang G. H. Deng S. Q. Li C. Liu Y. D. Chen L. Z. (2012). Damage to DNA caused by UV-B radiation in the desert cyanobacterium *Scytonema javanicum* and the effects of exogenous chemicals on the process. Chemosphere 88, 413–417. doi: 10.1016/j.chemosphere.2012.02.056, PMID: 22436589

[ref128] Wang G. H. Hao Z. J. Huang Z. B. Hu C. X. Liu Y. D. (2010). Raman spectroscopic analysis of a desert cyanobacterium *Nostoc* sp. in response to UV-B radiation. Astrobiology 10, 783–788. doi: 10.1089/ast.2009.0407, PMID: 21087158

[ref129] Wang X. P. Li X. R. Xiao H. L. (2005). Evolution characteristics of the artificially re-vegetated shrub ecosystem of arid and semi-arid sand dune area. Acta Ecol. Sin. 25, 1974–1980.

[ref130] Wang W. B. Liu Y. D. Li D. H. Hu C. X. Rao B. Q. (2008b). Feasibility of cyanobacterial inoculation for biological soil crusts formation in desert area. Soil Biol. Biochem. 41, 926–929. doi: 10.1016/j.soilbio.2008.07.001

[ref131] Wang W. B. Yang C. Y. Tang D. S. (2007). Effects of sand burial on biomass, chlorophyll fluorescence and extracellular polysaccharides of man-made cyanobacterial crusts under experimental conditions. Sci. China C Life Sci. 50, 530–534. doi: 10.1007/s11427-007-0051-z, PMID: 17653676

[ref132] Wang X. Q. Zhang Z. M. Jiang J. Yang W. K. Guo H. X. Hu Y. F. (2009b). Effects of spring-summer grazing on longitudinal dune surface in southern gurbantunggut desert. J. Geogr. Sci. 19, 299–308. doi: 10.1007/s11442-009-0299-2

[ref133] Wang H. Zhang G. Liu F. Geng R. Wang L. (2017). Temporal variations in infiltration properties of biological crusts covered soils, on the loess plateau of China. Catena 159, 115–125. doi: 10.1016/j.catena.2017.08.009

[ref134] Wang X. Q. Zhang Y. M. Zhang W. M. Han Z. W. (2009a). Comparison of erodibility on four types biological crusts in gurbantunggut desert from wind tunnel experiments. J. Arid Land Stud. 19, 237–240.

[ref135] Wei J. C. (2005). Biocarpet engineering using microbiotic crust for controlling sand. Arid Zone Res. 22, 287–288.

[ref136] West N. E. (1990). Structure and function of microphytic soil crusts in wildland ecosystems of arid to semi-arid regions. Adv. Ecol. Res. 20, 179–223. doi: 10.1016/S0065-2504(08)60055-0

[ref137] Wu Z. Y. (2021). Vegetation of China (English Version). China Agricultural University Press, Beijing.

[ref138] Wu H. Y. Gao K. S. Villafane V. E. (2005). Effects of solar UV radiation on morphology and photosynthesis of filamentous cyanobacterium *Arthrospira platensis*. Appl. Environ. Microbiol. 71, 5004–5013. doi: 10.1128/AEM.71.9.5004-5013.2005, PMID: 16151080 PMC1214621

[ref139] Wu L. Lan S. B. Zhang D. L. Hu C. X. (2012). Functional reactivation of photosystem II in lichen soil crusts after long-term desiccation. Plant Soil 369, 177–186. doi: 10.1007/s11104-012-1563-2

[ref140] Wu L. Lan S. B. Zhang D. L. Hu C. X. (2013). Recovery of chlorophyll fluorescence and CO_2_ exchange in lichen soil crusts after rehydration. Eur. J. Soil Biol. 55, 77–82. doi: 10.1016/j.ejsobi.2012.12.006

[ref141] Wu L. Lei Y. P. Lan S. B. Hu C. X. (2017). Photosynthetic recovery and acclimation to excess light intensity in the rehydrated lichen soil crusts. PLoS One 12:e0172537. doi: 10.1371/journal.pone.0190116, PMID: 28257469 PMC5336202

[ref142] Wu Y. S. Li X. R. Hasi E. D. Yin R. P. Liu T. J. (2020). Surface roughness response of biocrust-covered soil to mimicked sheep trampling in the mu us sandy land, northern China. Geoderma 313:114143. doi: 10.1016/j.geoderma.2019.114146

[ref143] Wu Q. F. Liu H. J. (2008). Effect of range fire on nitrogen fixation of *Collema tenaxin* a semiarid grassland of Inner Mongolia, China. J. Plant Ecol. 32, 908–913. doi: 10.3773/j.issn.1005-264x.2008.04.020

[ref144] Wu N. Zhang Y. M. Downing A. (2009). Comparative study of nitrogenase activity in different types of biological soil crusts in the gurbantunggut desert, northwestern China. J. Arid Environ. 73, 828–833. doi: 10.1016/j.jaridenv.2009.04.002

[ref145] Wu L. Zhang G. Lan S. Zhang D. L. Hu C. X. (2014). Longitudinal photosynthetic gradient in crust lichens’ thalli. Microb. Ecol. 67, 888–896. doi: 10.1007/s00248-014-0366-9, PMID: 24477924

[ref146] Wu L. Zhang Y. M. Zhang J. Downing A. (2015). Precipitation intensity is the primary driver of moss crust-derived CO_2_ exchange: implication for soil C balance in a temperate desert of northern China. Eur. J. Soil Biol. 67, 27–34. doi: 10.1016/j.ejsobi.2015.01.003

[ref147] Xiao B. Hu K. L. (2017). Moss-dominated biocrusts decrease soil moisture and result in the degradation of artificially planted shrubs under semiarid climate. Geoderma 291, 47–54. doi: 10.1016/j.geoderma.2017.01.009

[ref148] Xiao B. Hu K. L. Ren T. S. Li B. G. (2016). Moss-dominated biological soil crusts significantly influence soil moisture and temperature regimes in semiarid ecosystems. Geoderma 263, 35–46. doi: 10.1016/j.geoderma.2015.09.012

[ref149] Xiao B. Sun F. H. Yao X. M. Hu K. L. Kidron G. J. (2019). Seasonal variations in infiltrability of moss-dominated biocrusts on aeolian sand and loess soil in the Chinese loess plateau. Hydrol. Process. 33, 2449–2463. doi: 10.1002/hyp.13484

[ref150] Xiao B. Wang Q. H. Zhao Y. G. Shao M. A. (2011). Artificial culture of biological soil crusts and its effects on overland flow and infiltration under simulated rainfall. Appl. Soil Ecol. 48, 11–17. doi: 10.1016/j.apsoil.2011.02.006

[ref151] Xiao B. Zhao Y. G. Shao M. A. (2010). Characteristics and numeric simulation of soil evaporation in biological soil crusts. J. Arid Environ. 74, 121–130. doi: 10.1016/j.jaridenv.2009.06.013

[ref152] Xiao B. Zhao Y. G. Wang Q. H. Li C. (2015). Development of artificial moss-dominated biological soil crusts and their effects on runoff and soil water content in a semi-arid environment. J. Arid Environ. 117, 75–83. doi: 10.1016/j.jaridenv.2015.02.017

[ref153] Xie Z. M. Wang Y. X. Liu Y. D. Liu Y. M. (2009). Ultraviolet-B exposure induces photo-oxidative damage and subsequent repair strategies in a desert cyanobacterium *Microcoleus vaginatus* Gom. Eur. J. Soil Biol. 45, 377–382. doi: 10.1016/j.ejsobi.2009.04.003

[ref154] Xu S. J. Yin C. S. He M. Wang Y. (2008). A technology for rapid reconstruction of moss-dominated soil crusts. Environ. Eng. Sci. 25, 1129–1137. doi: 10.1089/ees.2006.0272

[ref155] Xue L. G. Zhang Y. Zhang T. G. An L. Wang X. (2005). Effects of enhanced ultraviolet-B radiation on algae and cyanobacteria. Crit. Rev. Microbiol. 31, 79–89. doi: 10.1080/10408410590921727, PMID: 15986833

[ref156] Yang H. Y. Liu C. Z. Liu Y. M. Xing Z. S. (2018). Impact of human trampling on biological soil crusts determined by soil microbial biomass, enzyme activities and nematode communities in adesert ecosystem. Eur. J. Soil Biol. 87, 61–71. doi: 10.1016/j.ejsobi.2018.05.005

[ref157] Yang J. Wei J. C. (2014). Desert lichens in shapotou region of tengger desert and bio-carpet engineering. Mycosystema 33, 1025–1035. doi: 10.13346/j.mycosystema.130150

[ref158] Yin B. F. Zhang Y. M. (2016). Physiological regulation of *Syntrichia caninervis* mitt. In different microhabitats during period of snow in the gurbantunggut desert, northwestern China. J. Plant Physiol. 194, 13–22. doi: 10.1016/j.jplph.2016.01.015, PMID: 26948275

[ref159] Zhang Y. M. (2005). The microstructure and formation of biological soil crusts in their early developmental stage. Chin. Sci. Bull. 50, 117–121. doi: 10.1007/BF02897513

[ref160] Zhang Y. M. Chen J. Wang L. Wang H. O. Gu Z. H. (2007). The spatial distribution patterns of biological soil crusts in the gurbantunggut desert, northern Xinjiang, China. J. Arid Environ. 68, 599–610. doi: 10.1016/j.jaridenv.2006.06.012

[ref161] Zhang Z. S. Chen Y. L. Xu B. X. Li X. R. (2015). Topographic differentiations of biological soil crusts and hydraulic properties in fixed sand dunes, tengger desert. J. Arid. Land 7, 205–215. doi: 10.1007/s40333-014-0048-y

[ref162] Zhang X. K. Dong X. W. Liang W. J. (2010). Spatial distribution of soil nematode communities in stable and active sand dunes of horqin sandy land. Arid Land Res. Manag. 24, 68–80. doi: 10.1080/15324980903439321

[ref163] Zhang Z. S. Dong X. J. Liu Y. B. Li X. R. Jia R. L. Hu Y. G.. (2012b). Soil oxidases recovered faster than hydrolases in a 50-year chronosequence of desert revegetation. Plant Soil 358, 275–287. doi: 10.1007/s11104-012-1162-2

[ref164] Zhang P. Li X. R. He M. Z. (2012a). Effects of wintertime low temperature and simulated warming on nitrogen-fixing activity of soil biocrusts. Chin. J. Ecol. 31, 1653–1658.

[ref165] Zhang B. C. Li R. H. Xiao P. Su Y. Zhang Y. (2016a). Cyanobacterial composition and spatial distribution based on pyrosequencing data in the gurbantunggut desert, northwestern China. J. Basic Microbiol. 56, 308–320. doi: 10.1002/jobm.201500226, PMID: 26479723

[ref166] Zhang Z. S. Liu L. C. Li X. R. Zhang J. G. He M. Z. Tan H. J. (2008). Evaporation properties of a revegetated area of the tengger desert, North China. J. Arid Environ. 72, 964–973. doi: 10.1016/j.jaridenv.2007.11.010

[ref167] Zhang T. Liu M. Wang Y. Y. Wei X. L. Wei J. C. (2017). Two new species of Endocarpon (*Verrucariaceae, Ascomycota*) from China. Sci. Rep. 7:7193. doi: 10.1038/s41598-017-07778-5, PMID: 28775314 PMC5543127

[ref168] Zhang Y. M. Wang H. L. Wang X. Q. Yang W. K. Zhang D. Y. (2006). The microstructure of microbiotic crust and its influence on wind erosion for a sandy soil surface in the gurbantunggut desert of northwestern China. Geoderma 132, 441–449. doi: 10.1016/j.geoderma.2005.06.008

[ref169] Zhang J. Zhang Y. M. (2014). Diurnal variation of chlorophyll fluorescence and CO_2_ exchange of biological soil crusts in different successional stages in the gurbantunggut desert of northwestern China. Ecol. Res. 29, 289–298. doi: 10.1007/s11284-013-1122-1

[ref170] Zhang J. Zhang Y. M. Alison D. Chen J. H. Zhou X. B. Zahng B. C. (2009b). The influence of biological soil crusts on dew deposition in gurbantunggut desert, northwestern China. J. Hydrol. 379, 220–228. doi: 10.1016/j.jhydrol.2009.09.053

[ref171] Zhang B. C. Zhang Y. M. Downing A. Niu Y. (2011a). Distribution and composition of cyanobacteria and microalgae associated with biological soil crusts in the gurbantunggut desert, China. Arid Land Res. Manag. 25, 275–293. doi: 10.1080/15324982.2011.565858

[ref172] Zhang J. Zhang Y. M. Downing A. Wu N. Zhang B. C. (2011b). Photosynthetic and cytological recovery on remoistening *Syntrichia caninervis* mitt., a desiccation-tolerant moss from northwestern China. Photosynthetica 49, 13–20. doi: 10.1007/s11099-011-0002-6

[ref173] Zhang B. C. Zhang Y. M. Su Y. G. Zahng J. Z. Zhang J. (2013). Responses of microalgal-microbial biomass and enzyme activities of biological soil crusts to moisture and inoculated *Microcoleus vaginatus* gradients. Arid Land Res. Manag. 27, 216–230. doi: 10.1080/15324982.2012.754514

[ref174] Zhang B. C. Zhang Y. M. Zhao J. C. Chen R. Y. (2009a). Microalgal species variation at different successional stages in biological soil crusts of the gurbantunggut desert, northwestern China. Biol. Fertil. Soils 45, 539–547. doi: 10.1007/s00374-009-0364-0

[ref175] Zhang Y. M. Zhou X. B. Yin B. F. Downing A. (2016b). Sensitivity of the xerophytic moss *Syntrichia caninervis* to prolonged simulated nitrogen deposition. Ann. Bot. 117, 1153–1161. doi: 10.1093/aob/mcw058, PMID: 27085182 PMC4904175

[ref176] Zhang B. C. Zhou X. B. Zhang Y. M. (2014). Responses of microbial activities and soil physical-chemical properties to the successional process of biological soil crusts in the gurbantunggut desert, Xinjiang. J. Arid. Land 7, 101–109. doi: 10.1007/s40333-014-0035-3

[ref177] Zhao H. L. Guo Y. R. Zhou R. L. Drake S. (2011). The effects of plantation development on biological soil crust and topsoil properties in a desert in northern China. Geoderma 160, 367–372. doi: 10.1016/j.geoderma.2010.10.005

[ref178] Zhao R. M. Hui R. Wang Z. R. An L. Z. (2016a). Winter snowfall can have a positive effect on photosynthetic carbon fixation and biomass accumulation of biological soil crusts from the gurbantunggut desert, China. Ecol. Res. 31, 251–262. doi: 10.1007/s11284-016-1335-1

[ref179] Zhao Y. Jia R. L. Teng J. L. (2017). Responses of biological soil crust coverage to wind-blown sand burial during the succession of artificial sand-fixing vegetation in the tengger desert, northern China. Acta Ecol. Sin. 37, 6138–6148. doi: 10.5846/stxb201606231227

[ref180] Zhao Y. Jia R. L. Wang J. (2019). Towards stopping land degradation in drylands: water-saving techniques for cultivating biocrusts in situ. Land Degrad. Dev. 30, 2336–2346. doi: 10.1002/ldr.3423

[ref181] Zhao Y. Li X. R. Zhang Z. S. Hu Y. G. Chen Y. L. (2014b). Biological soil crusts influence carbon release responses following rainfall in a temperate desert, northern China. Ecol. Res. 29, 889–896. doi: 10.1007/s11284-014-1177-7

[ref182] Zhao L. N. Liu Y. B. Wang Z. R. Yuan S. W. Qi J. H. Zhang W. L.. (2020b). Bacteria and fungi differentially contribute to carbon and nitrogen cycles during biological soil crust succession in arid ecosystems. Plant Soil 447, 379–392. doi: 10.1007/s11104-019-04391-5

[ref183] Zhao L. N. Liu Y. B. Yuan S. W. Li Z. H. Sun J. Y. Li X. R. (2020a). Development of archaeal communities in biological soil crusts along a revegetation chronosequence in the tengger desert, north Central China. Soil Tillage Res. 196:104443. doi: 10.1016/j.still.2019.104443

[ref184] Zhao Y. G. Qin N. T. Weber B. Xu M. X. (2014a). Response of biological soil crusts to raindrop erosivity and underlying influences in the hilly loess plateau region, China. Biodivers. Conserv. 23, 1669–1686. doi: 10.1007/s10531-014-0680-z

[ref185] Zhao L. J. Xiao H. L. Cheng G. D. Liu X. H. Yang Q. Yang Q.. (2010b). Correlation between δ^13^ C and δ^15^ N in C_4_ and C_3_ plants of natural and artificial sand-binding microhabitats in the tengger desert of China. Ecol. Inform. 5, 177–186. doi: 10.1016/j.ecoinf.2009.08.004

[ref186] Zhao Y. G. Xu M. X. (2013). Runoff and soil loss from revegetated grasslands in the hilly loess plateau region, China: influence of biocrust patches and plant canopies. J. Hydrol. Eng. 18, 387–393. doi: 10.1061/(ASCE)HE.1943-5584.0000633

[ref187] Zhao Y. G. Xu M. X. Belnap J. (2010a). Potential nitrogen fixation activity of different aged biological soil crusts from rehabilitated grasslands of the hilly loess plateau, China. J. Arid Environ. 74, 1186–1191. doi: 10.1016/j.jaridenv.2010.04.006

[ref188] Zhao Y. Xu W. W. Wang N. (2021). Effects of covering sand with different soil substrates on the formation and development of artificial biocrusts in a natural desert environment. Soil Tillage Res. 213:105081. doi: 10.1016/j.still.2021.105081

[ref189] Zhao Y. Zhang Z. S. Hu Y. G. Chen Y. L. (2016b). The seasonal and successional variations of carbon release from biological soil crust-covered soil. J. Arid Environ. 127, 148–153. doi: 10.1016/j.jaridenv.2015.11.012

[ref190] Zhi D. J. Nan W. B. Ding X. X. Li H. Y. (2009). Soil nematode community succession in stabilised sand dunes in the tengger desert, China. Soil Res. 47, 508–517. doi: 10.1071/SR08196

[ref191] Zhou X. J. Ke T. Li S. X. Deng S. Q. An X. L. Ma X.. (2020). Induced biological soil crusts and soil properties varied between slope aspect, slope gradient and plant canopy in the hobq desert of China. Catena 190:104559. doi: 10.1016/j.catena.2020.104559

[ref192] Zhou X. B. Zhang Y. M. (2014). Temporal dynamics of soil oxidative enzyme activity across a simulated gradient of nitrogen deposition in the gurbantunggut desert, northwestern China. Geoderma 213, 261–267. doi: 10.1016/j.geoderma.2013.08.030

[ref193] Zhou X. B. Zhang Y. M. Yin B. F. (2016). Divergence in physiological responses between cyanobacterial and lichen crusts to a gradient of simulated nitrogen deposition. Plant Soil 399, 121–134. doi: 10.1007/s11104-015-2687-y

[ref194] Zhou X. B. Zhao Y. G. Belnap J. Zhang B. C. Bu C. F. Zhang Y. M. (2020). Practices of biological soil crust rehabilitation in China: experiences and challenges. Restor. Ecol. 28, 45–55. doi: 10.1111/rec.13148

